# On-chip 3D printing of metal microinductors for radio frequency electronics

**DOI:** 10.1038/s41467-026-77493-1

**Published:** 2026-09-08

**Authors:** Alessandro Paghi, Farbod Abazar, Valeria Lazzoni, Angelica Masi, Sabrina Rotundo, Danilo Brizi, Agostino Monorchio, Giuseppe Barillaro

**Affiliations:** 1 Dipartimento di Ingegneria dell’Informazione, Università di Pisa, Pisa, Italy; 2 NEST, Scuola Normale Superiore and Istituto Nanoscienze-CNR, Pisa, Italy; 3 Centro per l’Integrazione della Strumentazione Scientifica dell’Università di Pisa (CISUP), Pisa, Italy

**Keywords:** Electrical and electronic engineering, Electronic devices

## Abstract

On-chip inductors are essential components of radio-frequency integrated circuits, yet their large footprint and limited scalability constrain chip miniaturization and increase manufacturing costs. Here we show a method for integrating three-dimensional metal microinductors directly onto chip contact pads using high-precision electrodeposition. The resulting freestanding coils extend above the chip surface, decoupling their inductance from the planar silicon area they occupy. We fabricate copper microsolenoids with different geometries directly on test chips and characterize their electrical performance over a broad frequency range. The microsolenoids exhibit inductance in the nanohenry range, quality factors of up to 18, and retain inductive behaviour up to 15 GHz, in agreement with electromagnetic simulations. The process is maskless and can be applied after standard chip fabrication without modifying the underlying circuit design, providing a route towards smaller, more scalable, and potentially lower-cost radio-frequency integrated circuits.

## Abstract (web summary)

Researchers use precision electrodeposition to 3D print copper microinductors directly onto chip contact pads. By extending above the chip surface, the coils decouple inductance from planar silicon area while maintaining inductive operation up to 15 GHz.

## Introduction

On-chip inductors are essential components of radio frequency integrated circuits (RF-ICs), enabling key functionalities in systems, such as low-noise amplifiers (LNAs), mixers, and oscillators that underpin modern wireless communication technologies and the Internet of Things (IoT)^1^. As RF systems evolve toward higher frequencies and greater integration density, the performance and scalability of on-chip inductors become increasingly critical.

**Fig. 1:**
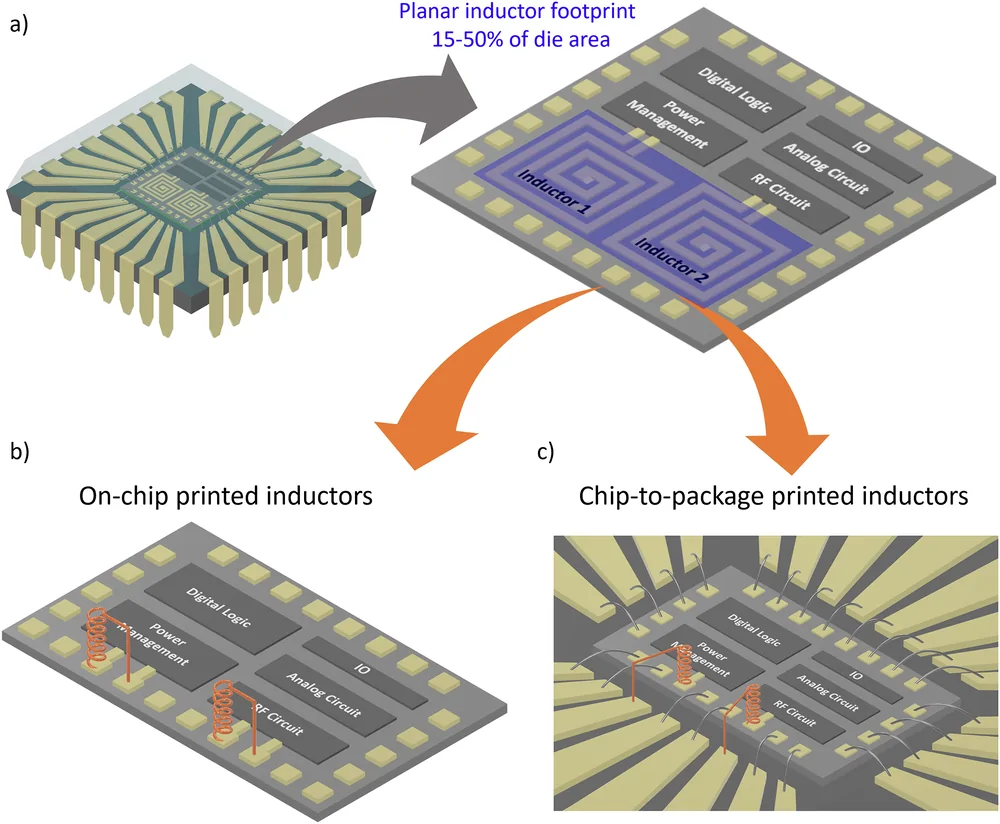
Concept of zero-footprint on-chip metal microinductors enabled by 3D printing. **a** Conventional on-chip inductors occupy a significant portion of the planar silicon area in radio-frequency integrated circuits (RF-ICs), typically 15–50% of the total die area, depending on the chip architecture and technology node^3^). Three-dimensional microinductors are directly printed onto chip contact pads (**b**) or across chip-to-package interconnects (**c**), eliminating the need for additional dedicated planar silicon area. This architecture has the potential to recover 15–50% of the silicon area typically occupied by planar inductors, enabling increased integration density, additional active circuitry, or reduced die size, depending on the RF-IC design.

Despite their importance, on-chip inductors represent one of the main bottlenecks in RF-IC design owing to their large footprint and limited scalability. Typical performance requirements include inductance values in the 1–10 nH range, quality factors (*Q*) exceeding 10 at the operating frequency, and self-resonance frequencies above 10 GHz^2^. Achieving these specifications inherently requires large planar structures, and inductors typically occupy 15–50% of the total die area, depending on the technology node and RF circuit architecture^3^. For example, inductors account for approximately 20% of the silicon area in LNAs, 18% in band-pass filters, and up to 40% in transformer-based layouts^3^. Since semiconductor fabrication cost increases superlinearly with die area due to yield considerations, the silicon area devoted to passive inductors has a disproportionate impact on both manufacturing cost and integration density. Unlike transistors and interconnects, which benefit from continuous downscaling, the footprint of conventional on-chip planar inductors is fundamentally constrained by electromagnetic considerations and therefore does not scale proportionally with CMOS technology nodes. Consequently, the inductor footprint has emerged as one of the principal barriers to further RF-IC miniaturization. Figure 1a illustrates a representative RF-IC layout, highlighting the substantial fraction of silicon area conventionally occupied by inductors. A simplified package representation is used to highlight the placement of the inductor on the chip.

Numerous approaches have been proposed to address these challenges, including layout optimization^4,5^, micro-electromechanical system (MEMS) technologies^6,7^, three-dimensional self-rolled-up^8,9^ and vertical-stacked architectures^10,11^, as well as the use of magnetic cores and dielectrics^2,12^ and the incorporation of carbon nanotube bundles and graphene multilayers^3,13^. As summarized in Table S1, these approaches achieve different trade-offs between inductance, quality factor, fabrication complexity, and footprint. While many improve inductance density or quality factor, they generally preserve the fundamental trade-off between inductive performance and dedicated planar silicon area, and often require increasingly complex fabrication processes.

In this context, additive manufacturing techniques, such as 3D printing, offer opportunities to realize three-dimensional structures beyond the constraints of planar fabrication^14,15^. Notable approaches include two-photon polymerization^16^, multi-photon lithography^17^, and ice lithography^18^. However, most high-resolution techniques rely on polymer-based processes that require subsequent metallization, limiting conductivity, scalability, and compatibility with standard microelectronic platforms. As further highlighted in Table S1, direct 3D printing of fully metallic on-chip microinductors has remained largely unexplored despite its potential to decouple inductive performance from the planar silicon-area budget.

Here, we introduce an architectural solution to the longstanding footprint limitation of planar inductors through the on-chip integration of three-dimensional copper microinductors for high-frequency applications that require no additional planar silicon area, enabled by in situ electrodeposition with sub-micrometer resolution. By directly printing freestanding metal-air microsolenoids onto chip contact pads (Fig. 1b) or across chip-to-package interconnects (Fig. 1c), the proposed approach decouples inductance from the planar silicon-area budget, enabling recovery of the silicon area conventionally occupied by inductors for increased integration density, additional active circuitry, or reduced die size. We demonstrate the fabrication of copper microsolenoids with varying geometrical parameters directly on the test chip and perform comprehensive electrical characterization. The microsolenoids exhibit inductance values in the nH range and quality factors up to 18 in the GHz regime, in agreement with electromagnetic simulations, with extended measurements confirming inductive behavior up to 15 GHz.

## Results and discussion

**Fig. 2:**
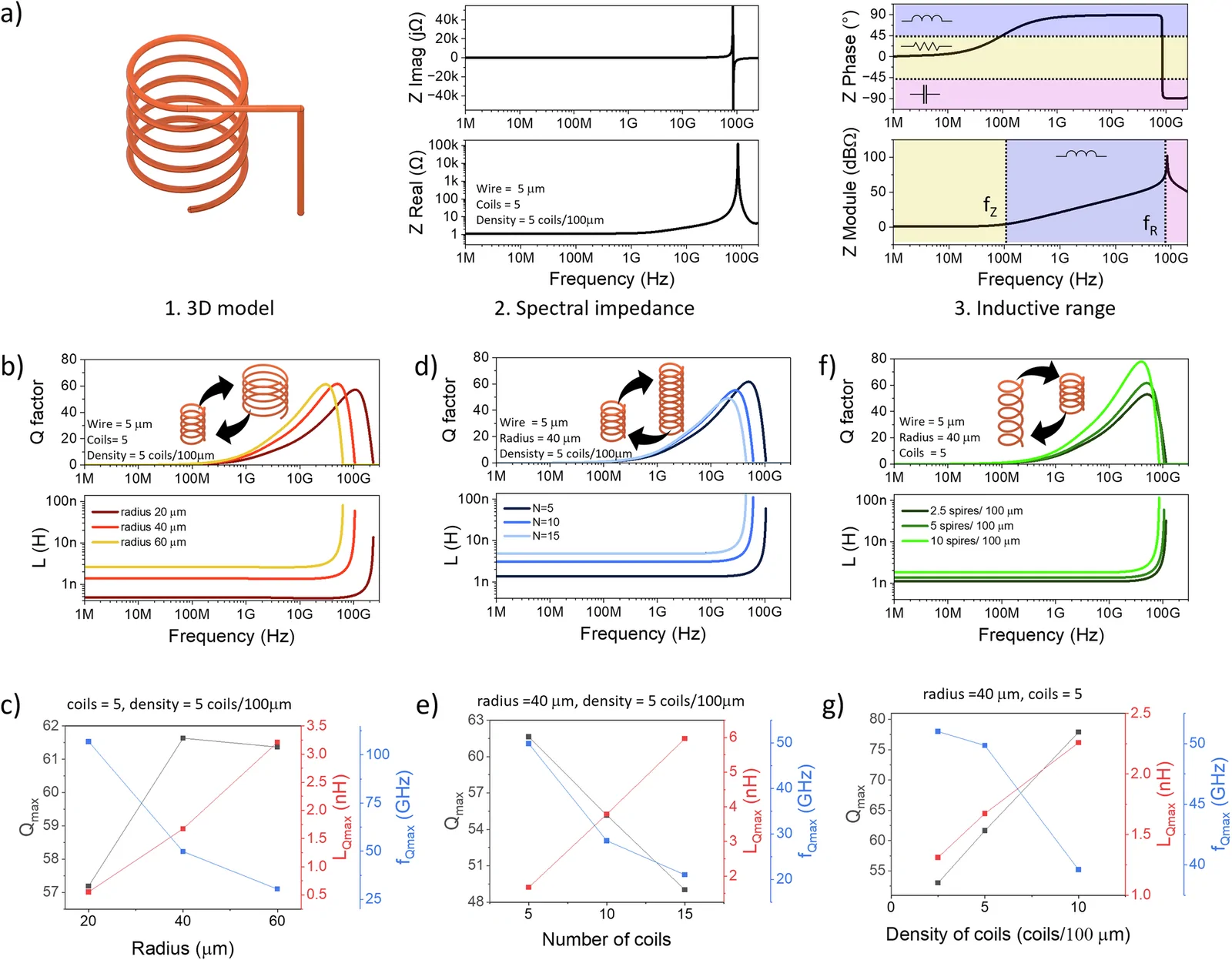
3D full-wave electromagnetic simulations of printed metal microsolenoids. **a** Electromagnetic simulation of a representative microsolenoid: 1. 3D model of the microsolenoid; 2. real and imaginary parts of the impedance as a function of frequency; 3. magnitude and phase of the impedance over frequency, with the inductive range highlighted in blue and the resistive and capacitive regions in yellow and pink, respectively. Simulated quality factor (*Q*) and inductance (*L*) as a function of frequency for microsolenoids with varying coil radius (**b**), number of turns (**d**), and coil density (**f**). **c**, **e**, **g** Summary of peak performance metrics corresponding to (**b**, **d**, **f**): maximum *Q* value (*Q*_max_, black), *L* value at *Q*_max_ (*L*_Qmax_, red), and frequency at *Q*_max_ (**f**_Qmax,_ blue).

We first conducted three-dimensional full-wave electromagnetic simulations to define the design space of the microsolenoids and investigate how the third dimension can be exploited to increase inductance and quality factor without increasing the dedicated planar silicon area required by conventional on-chip inductors. The simulations evaluate the frequency-dependent inductive behavior as a function of key geometrical parameters, namely coil radius, number of turns, and coil density, to identify design strategies for maximizing RF performance while preserving a compact planar footprint and to guide fabrication and characterization. A schematic of the simulated microsolenoid is shown in Fig. 2a-1. The parameter space explored included coil radii from 20 to 60 μm, number of turns from 5 to 15, and coil densities from 2.5 to 10 turns per 100 μm of height. The copper wire diameter was fixed at 5 μm, a value that is readily achievable with the printing system.

The commonly used long-solenoid approximation does not apply to these microsolenoids, as their length is comparable to their radius^19^. This leads to non-uniform magnetic field distributions and pronounced fringing effects, particularly for air-core inductors. In addition, at high frequencies, non-idealities, such as parasitic capacitance, radiation losses, skin effect, together with the limited number of turns, cause significant deviations from analytical models. Full 3D electromagnetic simulations are therefore required to accurately capture the inductive response and figures of merit of the printed microsolenoids, which are expected to deviate from the long-solenoid theory.

**Fig. 3:**
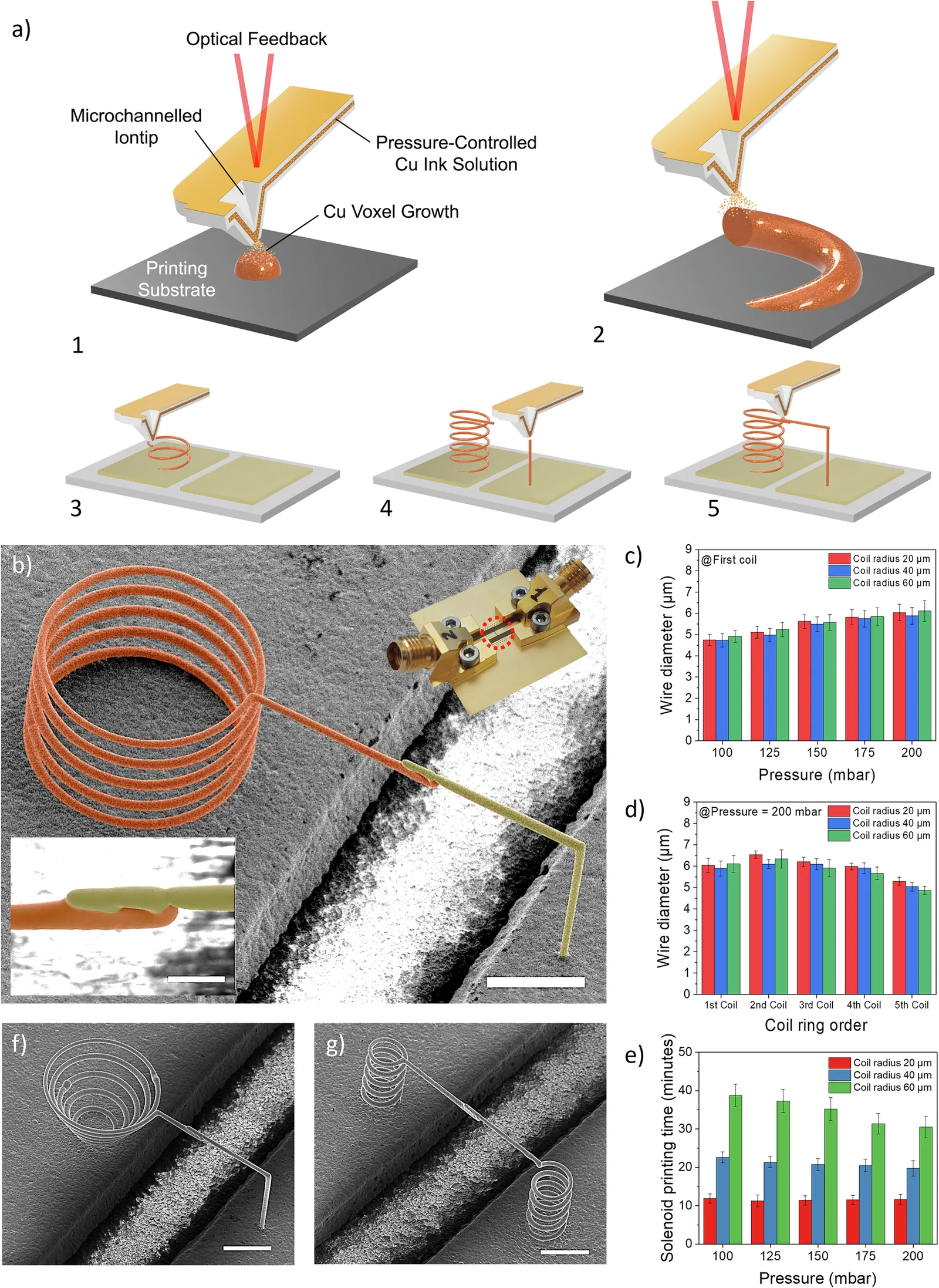
Fabrication and morphological characterization of on-chip 3D printed metal microinductors. **a** Fabrication process of Cu microinductors: 1. Cu ions delivered from the iontip are reduced at the substrate, forming a solid metal voxel; 2. the iontip is displaced to the next position; 3. a Cu microsolenoid is printed on one pad of the chip; a vertical Cu pillar is printed on the opposite pad (4) and connected to the microsolenoid via a horizontal metal wire (5). **b** False-color tilted-view SEM image of a representative microinductor consisting of a Cu solenoid (5 turns, radius 60 μm, wire diameter 5 μm, coil density 5 turns per 100 μm) printed onto a giCPW used for the electrical characterization; scale bar 50 μm. Bottom-left inset: detail of the interconnect between the two sides of the microinductor; scale bar 10 μm. Top-right inset: photograph of the giCPW with measurement connectors, highlighting the printing area (red dashed circle). Wire diameter as a function of extrusion pressure for the first coil (**c**) and as a function of coil order (**d**) for microsolenoids with different radii (*n* = 3 samples in (**c**) and *n* = 5 samples in (**d**) per radius). **e** Solenoid printing time as a function of extrusion pressure for microsolenoids with different radii (*n* = 3 samples per radius). Tilted-view SEM images of microinductors with variable-radius solenoids (**f**) and series-connected solenoids (**g**) printed onto a giCPW; scale bar 50 μm. Data in (**c**–**e**) are shown as mean value ± error bar representing the standard deviation.

Once the geometry of the microsolenoid is defined (Fig. 2a-1), the complex impedance $Z={Z}_{{real}}+j{Z}_{{imag}}$ is computed over the frequency range 1 MHz to 300 GHz (Fig. 2a-2), from which magnitude and phase are extracted (Fig. 2a-3). Figure 2a-2–3 show representative results of frequency-resolved impedance spectra for a Cu solenoid with 5 turns, radius of 40 μm, and height of 100 μm, exhibiting a self-resonance frequency *f*_*R*_ at 85 GHz. In the low-frequency regime (up to ~2 GHz), the resistive and inductive components remain relatively constant, indicating that skin effect and high-frequency losses become significant beyond this range. Accordingly, a lumped-element model adequately describes the printed solenoid behavior up to a few GHz.

The solenoid exhibits inductive behavior when the phase of *Z* exceeds 45°, corresponding to the frequency range between ${f}_{Z}=R/L$ and the self-resonance frequency ${f}_{R}=1/2\pi \sqrt{{LC}}$, where *L, R*, and *C* are the lumped parameters extracted from the impedance spectra (Fig. S1). Below *f*_*Z*_, the solenoid behaves predominantly as a resistor, while above *f*_*R*_ it becomes capacitive. The upper operational limit of the inductor is set by ${f}_{R}$, while the interval ${f}_{R}-{f}_{Z}$ defines the effective inductive operating range (Fig. 2a-3). Additional quantitative data on the lumped model parameters and corresponding inductive range are provided in Fig. S1.

The intrinsic properties of the microsolenoids are further evaluated through the inductance $L={Z}_{{imag}}/2\pi f$ and the quality factor $Q={Z}_{{imag}}/{Z}_{{real}}$, shown in Fig. 2b, d, f for varying coil radius, number of turns, and coil density. Increasing the coil radius leads to higher *L* and *Q* values at a given frequency due to the enhanced magnetic flux linkage and reduced relative resistive losses. However, this also lowers the self-resonance frequency and narrows the inductive range as a result of increased parasitic capacitance. Consequently, the peak quality factor *Q*_*max*_ saturates for radii above 40 μm (Fig. 2c). For a fixed coil radius (i.e., fixed planar footprint), the microsolenoid design exploits the third dimension to optimize inductive performance without increasing the dedicated planar area. Increasing the number of turns results in higher *L* values across the frequency range, owing to enhanced magnetic coupling and greater energy storage. However, the *Q* factor does not improve significantly; instead, its peak value decreases and shifts to lower frequencies due to increased parasitic capacitance and resistance associated with larger solenoid height (Fig. 2e). In contrast, increasing the coil density enhances both *L* and *Q* values, at a given frequency, by strengthening magnetic coupling and reducing radiative losses. This also increases *Q*_*max*,_ although at the expense of a reduced self-resonance frequency due to higher parasitic capacitance (Fig. 2g).

Across all configurations, *L* values remain in the nH range, peak *Q* values reach 60–80, and the maximum operating frequencies lie in the sub-THz range. Figure 2c–g summarize these trends by reporting the inductance ${L}_{{Q}_{\max }}$ and frequency ${f}_{{Q}_{\max }}$ values at which *Q* reaches its peak. For example, a solenoid with radius of 20 μm, 5 turns, and coil density of 5 turns per 100 μm exhibits a ${L}_{Q}{\max }\sim 0.56{{\mathrm{nH}}}$ and ${Q}_{\max } \sim 57$ at 100 GHz. Increasing the coil radius to 40 μm while keeping the number of turns and coil density unchanged increases ${L}_{{Q}_{\max }}$ to approximately $1.63{{\mathrm{nH}}}$, with comparable ${Q}_{\max }$ of about 6, although the peak shifts to a lower frequency of approximately 50 GHz. Alternatively, maintaining the same 40 μm radius (i.e., the same planar footprint) and increasing the coil density from 5 to 10 turns per 100 μm further increases ${L}_{{Q}_{\max }}$ to approximately $2.3{{\mathrm{nH}}}$ while increasing ${Q}_{\max }$ to about 80, with only a modest reduction in peak frequency to about 40 GHz.

This comparison highlights that, unlike conventional planar inductors where higher inductance is primarily achieved by increasing the footprint, the proposed microsolenoid architecture exploits the third dimension to further enhance inductive performance while preserving the planar footprint area.

We next fabricated the microsolenoids by room temperature, in situ direct metal printing based on localized electrodeposition of copper (Cu) with sub-micrometer resolution^20,21^ (Fig. 3a). Copper was selected for its high conductivity, cost-effectiveness, and compatibility with standard IC processes. The printing system employs a nanoscale nozzle (“iontip,” 300 nm) integrated at the apex of an atomic force microscopy (AFM) cantilever, through which a metal-ion-containing electrolyte (ink) is delivered via a microchannel connected to an internal reservoir. At the other end of the microchannel is a reservoir filled with the liquid ink. The iontip operates within a three-electrode electrochemical cell, where the conductive substrate acts as the working electrode. The ink and supporting electrolyte are those typically used in microelectronics for copper metal deposition of electrical interconnections and switches^22^. Upon application of extrusion pressure, the ink is delivered to the substrate, where metal ions are reduced, forming a solid copper voxel (Fig. 3a-1). The voxel grows until close proximity with the iontip is detected via optical beam deflection, triggering displacement of the tip to the next position and enabling voxel-by-voxel 3D growth (Fig. 3a-2). A sketch of the printing system is reported in Fig. S2.

**Fig. 4:**
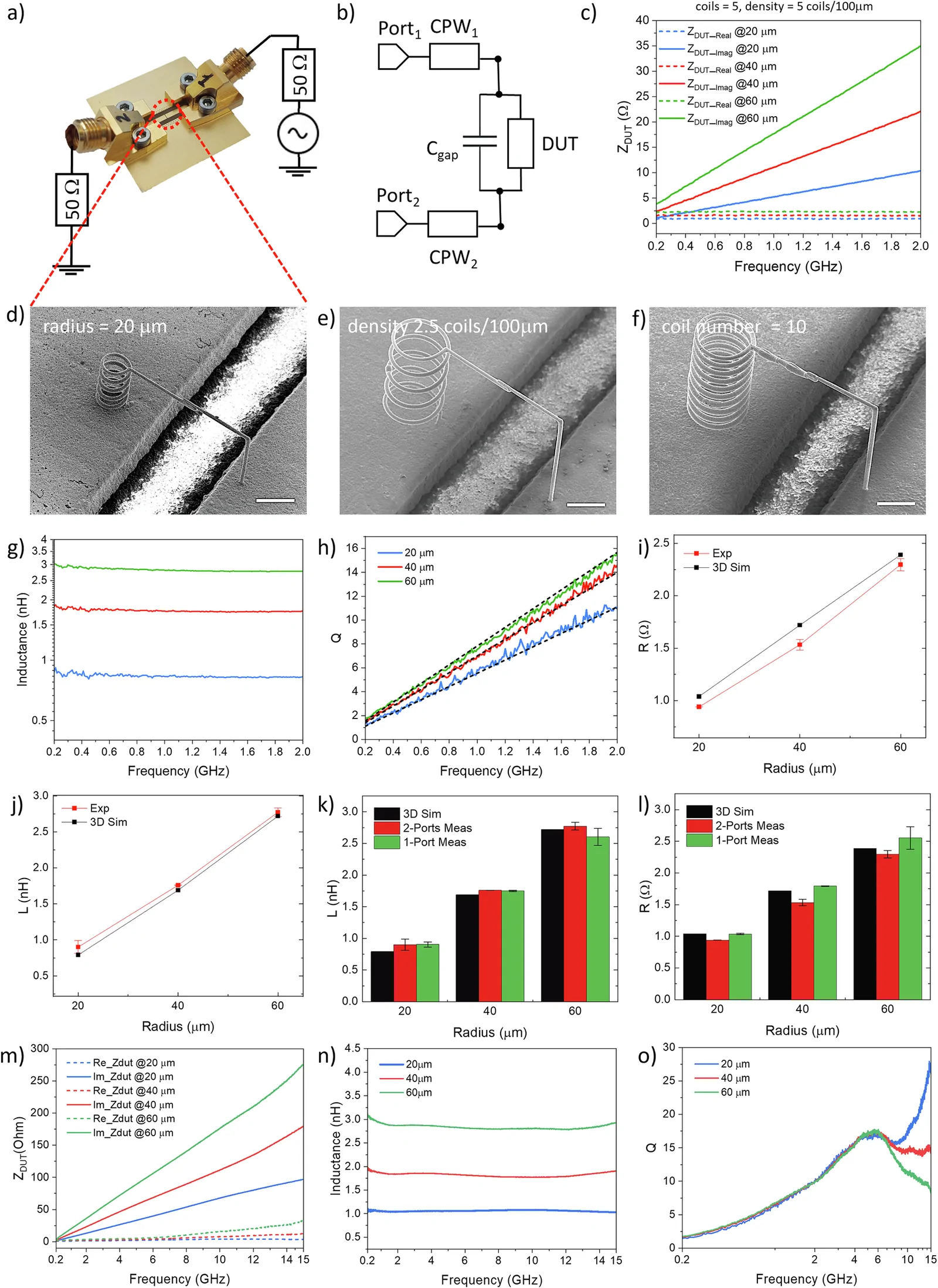
Electrical characterization of on-chip 3D-printed metal microinductors. **a** Photograph of the giCPW PCB with the microinductor printing area highlighted (red dashed circle). **b** Equivalent circuit model of the giCPW with integrated microinductors, used to extract the device-under-test impedance Z_DUT_ from measured S-parameters. **c** Frequency-resolved impedance spectra showing the real (dotted lines) and imaginary (solid lines) components of Z_DUT_ for microsolenoids with varying coil radii, measured up to 2 GHz. Tilted-view SEM images of representative printed microinductors: **d** radius 20 μm, 5 turns, 5 turns per 100 μm; **e** radius 40 μm, 5 turns, 2.5 turns per 100 μm; and **f** radius 40 μm, 10 turns, 10 turns per 100 μm. All structures are printed directly on the giCPW; scale bar 50 μm. **g**, **h** Frequency-resolved inductance (*L* = Imag(Z_DUT_)/*ω*) and quality factor (*Q* =  Imag(Z_DUT_)/Real(Z_DUT_)) extracted from (**c**) over the range 0.2–2 GHz. **i**, **j** Comparison between experimental and simulated lumped-model parameters (*L* and *R*) for microinductors with radii from 20 to 60 μm (*n* = 3 samples per radius). **k**, **l** Comparison of inductance and resistance values extracted from 2-port and 1-port measurements and from 3D electromagnetic simulations (*n* = 3 samples per radius). Data are presented as mean ± standard deviation. **m** Frequency-resolved impedance spectra showing the real (dotted lines) and imaginary (solid lines) components of Z_DUT_ for microinductors with radius of 20, 40, and 60 μm, measured up to 15 GHz. **n**, **o** Frequency-resolved inductance (*L* = Imag(Z_DUT_)/*ω*) and quality factor (*Q* = Imag(Z_DUT_)/Real(Z_DUT_)) extracted from (**m**) over the range 0.2–15 GHz. Data in (**i**, **j**, **k**, **l**) are shown as mean value ± error bar representing the standard deviation.

A Cu microsolenoid is first printed on one side of a gold-patterned substrate designed as a gap-interrupted grounded coplanar waveguide (giCPW) (Fig. 3a-3 and Fig. S3)^23^. A vertical Cu pillar is then printed on the opposite side and connected to the solenoid via a horizontal wire to establish electrical continuity (Fig. 3a-4–5). Figure 3b shows a representative false-color SEM image depicting a microinductor consisting of a three-dimensional copper solenoid (5 turns, 60 μm radius, 5 μm wire diameter) printed directly onto the giCPW. Additional structural and morphological details of the printed solenoid are given in Figs. S4 and S5. The printed structures exhibit sub-micrometer accuracy and low surface roughness (rms ~41 nm).

We explored microsolenoids with different radii of 20, 40, 60 μm, 5 and 9 turns, coil densities of 2.5 and 5 turns per 100 μm, and wire diameters of 4.5–6 μm (Fig. S6). The wire diameter scales linearly with extrusion pressure in the range 100–200 mbar, with good in-sample (CV% = 6.40%) and sample-to-sample (CV% = 7.54%) reproducibility (Fig. 3c and Fig. S7). A slight reduction in wire diameter of the microsolenoid is observed with increasing number of turns (up to ~15%, Fig. 3d). The printing time increases with coil radius, number of turns, and coil density, and decreases with increasing extrusion pressure (Fig. 3e).

The additive manufacturing approach offers substantial design flexibility, enabling a wide range of geometries beyond those explored here. Wire diameter can be increased by merging adjacent voxels to enhance mechanical robustness. For a given wire diameter, the achievable number of turns, coil density, and radius are primarily limited by the mechanical stability of the freestanding copper structures, governed by the effective Young’s modulus of the printed material.

Particular attention was devoted to optimize the electrical connection between the two parts of the inductor (the solenoid and the pillar) located on opposite sides of the giCPW. This was achieved through a 10 μm overlap of the horizontal wires connecting the upper coil and the pillar. The effect of the extrusion pressure (100–200 mbar) and wire-to-wire vertical distance (gap, 4–10 μm) on the connection quality was systematically investigated using SEM analysis (Figs. S8 and S9) and four-probe electrical measurements (Fig. S10). For gap values smaller than 5 μm, no connection is formed due to iontip collision with the previously printed wire, whereas for gaps larger than 9 μm, the separation is too large to enable bonding. Reliable electrical and mechanical connections are achieved for gap values between 6 and 8 μm. Within this range, increasing extrusion pressure enhances the contact area between the wires, leading to a corresponding reduction in electrical resistance (Fig. S10b, c). The measured resistance of the printed microinductors ranges from 0.6 to 1.8 Ω, depending on geometry and printing conditions. The extracted resistivity remains constant at 19 ± 2 nΩ m, corresponding to ~87% of the bulk copper conductivity, in agreement with previous reports^24^ (Fig. S10d). Optimal fabrication conditions were identified at an extrusion pressure of 200 mbar and a gap of 7 μm, and were adopted for all subsequent devices. The maximum current (*I*_max_) sustained before failure ranges from 130 to 200 mA and scales with the inverse square root of the coil radius, in agreement with theoretical expectations (Fig. S11a–c). The maximum power dissipation is approximately 20 mW, largely independent of coil radius (Fig. S11d). These results confirm the suitability of the printed microinductors for typical electronic applications^2^.

The printing process is robust and versatile, enabling a wide range of geometries, including solenoids with variable radius (Fig. 3f), series-connected solenoids (Fig. 3g), and concentric transformer configurations (Fig. S12).

We next performed electrical characterization of the Cu microinductors printed on the giCPW platform (Fig. 4). Representative SEM images of microinductors with varying geometrical parameters printed on the giCPW are shown in Fig. 4d–f. Two-port measurements were carried out to extract the scattering parameters (S-parameters) (Fig. 4a, b), from which the complex impedance of the device under test (${Z}_{{{{\rm{DUT}}}}}$) was obtained. This enabled the evaluation of frequency-dependent inductance, quality factor (*Q*), and resistance up to 2 GHz. The upper limit of this range is determined by the FR4 substrate used for the giCPW test platform, whose dielectric losses become significant at higher frequencies, as confirmed by simulations of the complete structure (Fig. S13).

Figure 4c shows the real and imaginary components of ${Z}_{{{{\rm{DUT}}}}}$ for microsolenoids with radii of 20, 40, and 60 μm (5 turns, 5 turns per 100 μm) over the 0.2–2 GHz range. The imaginary component increases linearly with frequency, consistent with inductive behavior, with a slope that increases with coil radius, reflecting higher inductance. The real component remains relatively constant and increases with radius due to the longer conductive path and associated series resistance. The extracted inductance and quality factor as a function of frequency are shown in Fig. 4g, h. Inductance values range from 0.8 to 3 nH depending on the solenoid radius and remain nearly constant across the measured frequency range. The quality factor increases with frequency and with coil radius, reaching values between 11 and 16 at 2 GHz, consistent with operation well below the self-resonance frequency.

These results confirm the inductive nature of the printed microsolenoids and support the use of a lumped-element electrical model up to 2 GHz. Direct fitting of S-parameter and Y-parameter data with an RLC_gap_ model (Figs. S14 and S15) yields inductance and resistance values in good agreement with both de-embedded measurements and 3D simulations (Fig. 4i, j), confirming the reliability of the fabrication and characterization approaches.

To further validate the extracted parameters, we performed complementary 1-port resonant measurements based on a giCPW terminated with an open circuit (Figs. S16 and S17). Adopting resonant transmission lines or waveguides is a well-known method for device-under-test (DUT) characterization^25,26^, serving as an alternative to conventional two-port transmissive measurements with a 50 Ω matched load. In this configuration, the resonance frequency of the giCPW shifts from ~5 GHz to 2–3 GHz upon integration of the microinductor, with the shift increasing with the inductance of the solenoid. Figure S16b shows a representative frequency-resolved spectrum of Z_11_ measured for microinductors with different radii (5 turns, 5 turns per 100 μm). Fitting of the measured ${Z}_{11}$ spectra using the same lumped-element model used for 2-port measurements shows excellent agreement, enabling independent extraction of *L* and *R* values.

Figure 4k, l compares the inductance and resistance obtained from 2-port and 1-port measurements with 3D simulations, showing strong agreement across all methods. The sample-to-sample variation (CV%) is below 7% for all solenoids tested. Consistent agreement between experimental and simulated inductance and resistance values is also observed for more complex geometries, including variable-radius and series-connected solenoids (Fig. S18), further confirming the robustness and reproducibility of the printing process.

To assess mechanical stability, selected microinductors were encapsulated in polydimethylsiloxane (PDMS) (Fig. S19). No significant variation in impedance spectra or extracted lumped parameters is observed before and after encapsulation, demonstrating that polymer embedding provides mechanical protection without degrading electrical performance.

Finally, the high-frequency response of the microsolenoids with different radii from 20 to 60 μm was investigated up to 15 GHz (Fig. 4m–o). Experimental measurements indicate excellent reliability of the high-frequency behavior over multiple devices (Figs. S20 and S21). The impedance spectra retain predominantly inductive behavior over the extended frequency range regardless of the microsolenoid radius, with the imaginary component increasing linearly with frequency, while the real component remains relatively low and exhibits a gradual increase due to frequency-dependent resistive losses (Fig. 4m). The inductance scales with the radius of the microsolenoids, remaining approximately constant over frequency and in agreement with the low frequency values, while the quality factor increases up to ~18 at ~5 GHz (Fig. 4n, o). The non-monotonic evolution of *Q* highlights that the upper frequency range is fundamentally limited by the dielectric losses and parasitic response of the FR4-based gap-interrupted coplanar waveguide used for electrical characterization, rather than by the printed microinductors themselves. These results demonstrate that the printed microinductors preserve inductive operation well into the multi-GHz regime.

A comparison with representative state-of-the-art air-core integrated inductor technologies is provided in Table S1, which summarizes their fabrication approach, geometry, inductance, quality factor, self-resonance frequency, footprint, and integration compatibility. The comparison shows that existing technologies achieve different trade-offs between electrical performance, footprint, and fabrication complexity. While some approaches report higher inductance or quality factor, these improvements are generally obtained at the expense of increased dedicated planar silicon area, more complex fabrication processes, or reduced compatibility with post-fabrication integration. In contrast, the present approach combines competitive RF performance with no additional dedicated planar silicon area, achieved by enabling direct printing of freestanding microsolenoids onto existing chip contact pads or interconnects. By exploiting the third dimension rather than increasing the planar footprint, this architecture decouples inductive performance from the planar silicon-area budget, providing a distinctive design strategy for future RF integrated circuits while remaining fully compatible with post-CMOS integration.

In conclusion, on-chip inductors remain a major bottleneck in RF-ICs because their large planar footprint and limited scalability constrain miniaturization, integration density, and manufacturing cost.

Here, we introduced an architectural solution to this longstanding limitation by enabling three-dimensional copper microinductors that require no additional dedicated planar silicon area. Using in situ electrodeposition with sub-micrometer resolution, freestanding metal-air microsolenoids were directly printed on a measurement chip, exploiting the third dimension as an additional degree of freedom for RF inductor design. The printed microinductors achieved inductance values in the nH range and quality factors up to 18 in the GHz regime, in agreement with full-wave electromagnetic simulations, with inductive behavior experimentally validated up to 15 GHz.

By decoupling inductance from the planar silicon-area budget, this approach enables increased inductive performance through three-dimensional design rather than increased silicon footprint.

The architecture is conceived as a post-fabrication integration strategy compatible with modern advanced semiconductor packaging technologies—including wafer-level packaging, fan-out packaging, redistribution-layer architectures, and system-in-package integration—which routinely accommodate non-planar structures and three-dimensional interconnects^27^. At the same time, practical aspects, such as package-level reliability, thermal management, and circuit-specific electromagnetic compatibility remain important considerations and will require dedicated investigation in future integrated RF-IC demonstrations.

Future work will focus on extending operation to higher frequencies through the use of low-loss substrates and custom IC platforms, as well as further improving performance through optimization of microsolenoid geometry, incorporation of functional magnetic materials, and enhancement of the metal printing process to reduce resistive losses and increase conductivity.

## Methods

### Materials and chemicals

Au-coated Si/SiO_2_ wafers, i.e., Si/SiO_2_(300 nm)/Cr(70 nm)/Ni:Vn 5 wt% (300 nm)/Au (50 nm), were provided by ST Microelectronics (Milan, Italy). Acetone (ACE, 99.8 wt%) and 2-propanol (IPA, 99.5 wt%) were purchased from CARLO ERBA Reagents (Milan, Italy). Hydrochloric acid (HCl, 37%), sulfuric acid (H_2_SO_4_, 95–97%), and hydrogen peroxide (H_2_O_2_, 30%) were purchased from Sigma-Aldrich (Germany). Iontip ink [Cu] (CuSO_4_ < 10 wt%, HCl < 0.002 wt%, and H_2_SO_4_ < 0.5 wt%) and iontip ink brighteners, i.e., diluted beta-naphthol, alkoxylated (<10 wt%), arylated polyethyleneimine (<0.001 wt%), and bis-(sodium sulfopropyl)-disulfide (<0.2 wt%) in purified water, were provided by EXADDON (Zürich, Switzerland). Iontips (aperture size = 300 nm, length = 200 µm, width = 36 µm, microchannel height = 1000 nm, spring constant = 2 N/m, tip air resonance = 78 kHz, and tip liquid resonance = 50 kHz) were purchased from EXADDON (Zürich, Switzerland). CERES 3D printer was purchased from EXADDON (Zürich, Switzerland). Rosenberger solderless PCB mount SMA connectors 02K80F-40ML5 were purchased from Digi-Key Electronics (USA). Aqueous solutions were prepared using deionized water (DIW, 18.2 MΩ × cm) filtered by Elix® (Merck Millipore).

### Microinductor design, simulation, and modeling

Microinductors featuring solenoids with different radii, number of turns, and turn densities were designed and simulated using a full-wave electromagnetic simulator (FEKO design suite, Altair Engineering Inc., Troy, MI, USA), based on the method of moments (MoM) numerical solver, to extrapolate the spectral impedance *Z* (*Z* = *Z*_real_ + j*Z*_imag_) over the frequency range 1 MHz to 300 GHz. The lumped model parameters of the simulated microinductors were then estimated as follows: lumped resistance (*R*) and inductance (*L*) values were extrapolated from *Z*_real_ and *Z*_imag_, respectively at frequencies far from the microinductor self-resonance; the lumped capacitance (*C*) value was obtained from the self-resonance frequency *f*_R_ = 1/2π√*LC*.

### Gap-interrupted ground coplanar waveguide (giCPW) design, simulation, and manufacturing

The microinductors were printed on a custom PCB designed as a gap-interrupted ground-backed coplanar waveguide (giCPW) with KiCAD (v5.1.7) software, leveraging both front and bottom copper (Cu) levels (Fig. S3). The giCPW was simulated using CST Studio Suite 2022.04 (Dassault Systemes, France) by resorting to the finite element method (FEM). The giCPW featured a length (L) of 12.3 mm and a width (W) of 1.1 mm interrupted by a gap (G) of 102 μm. We matched the impedance of the giCPW to *Z*_0_ = 50  Ω with a signal line-ground plane separation (S) of 1 mm. Higher-order resonance modes suppression was obtained with a double line ground vias placing (*V*_D1_ = 1.8 mm, *V*_D2_ = 2.15 mm, *V*_P_ = 0.4 mm, *V*_R_ = 0.2 mm)^28^. The external part of the giCPW was resized following the layout suggested for the solderless PCB-mount SMA connectors, provided by Rosenberger.

The giCPW PCB was manufactured exploiting the PCBWay *Normal process* (https://www.pcbway.com). FR-4 (thickness (H) of 0.6 mm, area of 25 mm × 25 mm, glass transition temperature of 130 °C) was used as the dielectric substrate of the PCB, with Cu layers on top and bottom featuring a minimum track/spacing of 102 μm and minimum via hole size of 200 μm (tenting vias technology). One oz Cu was chosen as the Cu finishing, and electroless nickel immersion gold (ENIG) as surface finishing, resulting in an overall metal thickness (T) of 35 μm.

The RF electrical behavior of the giCPW was evaluated as reported in Section *Measurements of Electrical Performance*. Simulations (Fig. S17) of the manufactured giCPW PCB were performed with the lumped element model reported in Fig. S17c, using AWR Design Environment 13. An ideal port (Port_1_, *Z*_0_ = 50 Ω) was used to feed the giCPW, whereas the other end is left open (open circuit). With the aim to simulate the transmission line, two CPW lumped elements (CPW_1,2_, *W* = 1.1 mm, *L* = 14.35 mm, *H* = 0.6 mm, *T* = 35 μm, *ε*_R_ = 4.3, tanδ_ε_ = 0.025) were implemented. Moreover, a lumped capacitor (*C*_gap_ = 34 fF) was exploited to reproduce the gap.

### Preparation of printing substrates

Au-coated SiO_2_(300-nm-thick)/Si substrates (25 mm × 25 mm) and giCPW PCBs were sonicated in ACE (5 min), IPA (5 min), and dried in N_2_. The signal line of the giCPW PCB was electrically connected with the top and bottom ground planes via Al/Si wire bonding. The wire bonding was eventually removed after printing of the microinductors.

### Printing of copper microinductors

The EXADDON CERES system was used for the 3D printing of copper microinductors. The system comprises: (i) a printing chamber, which is a 3-electrode electrochemical cell filled with an electrolyte solution (supporting electrolyte), including a graphite working electrode (W.E.), an Ag/AgCl reference electrode (R.E.), and a graphite counter electrode (C.E.); (ii) a modified atomic force microscope (AFM) equipped with an XYZ positioning stage with nanometer accuracy, optical force feedback, and an air pressure-driven liquid dispenser called iontip. The iontip extrudes the metal-salt-based printing solution (ink solution) near a conductive substrate immersed in the supporting electrolyte to obtain electrodeposited metal atoms. Figure S2 shows the block diagram schematic of the EXADDON CERES system.

The printing chamber was filled with 15 mL of the supporting electrolyte (H_2_SO_4_ 54 mM and HCl 0.5 mM in DIW), and a DC supply voltage of −0.5 V was applied by the potentiostat. Sufficient time was allowed for the current flowing through the electrochemical cell to stabilize, typically between 0.2 and 2 mA. Conductive printing substrates were then inserted inside the printing chamber with the top surface in contact with the working electrode, ensuring electrical contact. The ink solution was prepared by adding 20 μL of the ink brightener to 1 mL of [Cu] ink, and filtered with a 200 nm-size hydrophobic PTFE filter. The iontip’s reservoir was filled with 0.9 μL of the prepared ink solution, and the iontip was loaded in the system, following the CAPA “Prepare system” routine. The iontip was then characterized in terms of printing performance by manufacturing a calibration pillar with the following parameters: voxel height: 0.5 μm; section length: 20 μm; extrusion pressure: 160, 150, 140, 130, 120, 110, 100, 90, 80, 70, 60, 50, 40, 30, 20 mBar (Fig. S4). At the end of the process, a relationship between the printed Cu voxel diameter and the extrusion pressure applied to the iontip was obtained. This relationship was used as a calibration file to determine the thickness of subsequent printed structures.

The design of the printing structures was performed by using the Voxel Cloud Generator (VCG) script in MATLAB, provided by EXADDON. For Au-coated SiO_2_/Si substrates, the structures were printed as array elements on the exposed substrate surface. On the giCPW PCBs, the structures were printed in the designated area overlapping the gap, using the CAPA “Positioning routine.” At the end of the printing process, the printing substrate was extracted from the printing chamber and rinsed in ACE, then left to dry at room temperature. For giCPW PCBs, Al/Si wire bonds were removed to electrically isolate the signal line from the ground plane.

### Morphological characterization

Morphological characterization of on-chip 3D printed metal microinductors was carried out using a scanning electron microscope (JEOL JSM-6390) at an acceleration voltage of either 3 kV or 5 kV. Various magnifications were used to examine the dimensions of the microinductors as a function of the printing parameters. Surface roughness profiling of the printed structures was conducted using the ImageJ program with the additional SurfCharJ package. The root-mean-square (RMS) value was determined using a grayscale-based analysis of SEM images, a technique for estimating surface texture^29^. This method involves analyzing grayscale intensity variations that correspond to topographical features, once imaging parameters are known. Specifically, we employed ImageJ software equipped with the SurfCharJ plugin, which facilitates grayscale-based roughness extraction through gradient analysis and statistical texture mapping.

### Measurements of electrical performance

#### DC electrical behavior

DC electrical characterization of the 3D-printed microinductors was performed using a 4-wire measurement setup. A linear current sweep from 0 to 10 mA was applied while simultaneously measuring the voltage drop across the inductor. The DC resistance was extrapolated by fitting the slope of the measured VI curve. The giCPW PCB with inductor printed across the gap was placed under a probe-station (Everbeing probe station) provided with a high-magnification optical microscope and four precision manipulator probes, with two probes per side for contacting the ground-backed coplanar waveguide (GB-CPW). For the electrical breakdown measurements, the current sweep was extended to few hundreds of mA, and the corresponding voltage drop was recorded.

### Resistivity measurements

Resistivity characterization of the 3D-printed microinductors was carried out using the same 4-wire measurement setup described in the DC Electrical Behavior section. A linear current sweep from 0 to 10 mA was applied while simultaneously measuring the voltage drop across the inductor. The resistance was determined by linearly fitting the measured *I–V* curve. To calculate resistivity, the wire length and cross-sectional area were extracted from SEM images of the printed inductors (Fig. S6 and Fig. 2d). Given the observed variation in wire diameter along its length, this reduction was taken into account in the resistivity calculation. A Python script was used to calculate the resistivity value by combining the extracted resistance value with the non-uniform cross-sectional geometry data.

### RF electrical behavior

#### 2-port transmissive measurements

A Vector Network Analyzer (PNA-X N5267B, Keysight, Santa Rosa, CA, USA) was employed for 2-port S-parameter measurements over a frequency range from 100 MHz to 30 GHz. Calibration was performed using the Automatic Fixture Removal (AFR) technique with a through standard. The giCPW PCB was equipped with two solderless PCB-mount SMA connectors, enabling reproducible connections to the test fixture.

The measurement and analysis procedure can be summarized as follows:

Step 1. The S-parameters of the bare giCPW (without the device under test, DUT) were first measured to characterize the intrinsic response of the transmission line. These baseline measurements were used as a reference for subsequent de-embedding. From this initial dataset, a parasitic admittance model—primarily capturing the CPW gap capacitance (*C*_gap_)—was estimated and used to support the removal of fixture-related contributions from the DUT measurement.

Step 2. A second 2-port measurement was performed with the DUT (copper microinductor) printed across the CPW gap. The contribution of the giCPW parasitic admittance, including the previously estimated *C*_gap_, was removed from the total measured admittance. This procedure enabled isolation of the DUT’s intrinsic response and allowed extraction of its transfer impedance (Z_DUT_) via standard conversion from S-parameters.

Step 3. The impedance spectrum of the DUT was then modeled using a lumped-element RLC circuit, as illustrated in Fig. S14a. A direct nonlinear best-fitting of the measured S_11_ parameters was performed to extract the equivalent series resistance (*R*), inductance (*L*), and an updated parasitic capacitance (*C*_gap_). In parallel, the Y-parameters were retrieved from the measured S-parameters and independently fitted to the same equivalent circuit model, yielding consistent *R*, *L*, and *C* values and confirming the robustness of the extraction procedure (Fig. S15). The excellent agreement between experimental and best-fitting S_11_ and Y-parameter curves confirmed that the microinductor’s self-resonance frequency lies in the tens of GHz, supporting the use of a lumped-element approximation for modeling the DUT in the operational frequency band centered around 2 GHz.

#### 1-port resonant measurements

A Vector Network Analyzer (FieldFox series VNA N9918A, Keysight, Santa Rosa, CA, USA) was employed for 1-port measurements, covering a frequency range from 10 MHz to 20 GHz. The VNA calibration was conducted using a standard 2-port Short-Open-Load-Through (SOLT) procedure with the Keysight Cal Kit 85521A Type-3.5 mm (f) 50 Ω (from DC to 26.5 GHz). The giCPW PCBs, with and without the device under test (DUT), were equipped with two solderless PCB-mount SMA connectors. During measurement, Port 1 of the giCPW was actively fed, while Port 2 was terminated with a standard open circuit.

The characterization can be summarized as follows:

Step 1. The reflection coefficient (S_11_) of the bare giCPW (without DUT) was measured, and the corresponding impedance (*Z*_11_) was extracted. From this baseline response, the parasitic gap capacitance (*C*_gap_) of the giCPW was estimated via non-linear best-fitting of the measured *Z*_11_ with the model in Fig. S15c using AWR Design Environment 13, serving as an initial guess for subsequent de-embedding steps.

Step 2. A second measurement was conducted with the DUT (copper microinductor) printed across the CPW gap. By comparing this response with the baseline, the contribution of the DUT was isolated, and its impedance (*Z*_11_) was retrieved. Specifically, a nonlinear fitting procedure was carried out in AWR Design Environment 13, using a lumped-element RLC model (as illustrated in Fig. S16c) to extract the inductor’s equivalent series resistance (*R*), inductance (*L*), and an updated *C*_gap_. This model was supported by 3D full-wave electromagnetic simulations and 2-port measurements, both of which confirmed that the solenoid’s self-resonance lies in the tens of GHz, validating the use of a lumped-element approximation at the resonance of the giCPW—around 2 GHz.

In essence, the periodic quarter-wavelength resonance of the open-terminated giCPW is perturbed by the presence of the microsolenoid across the CPW gap. This perturbation, which is measurable with a high signal-to-noise ratio, can be analytically de-embedded and fitted to extract the electrical parameters of the inductor at the giCPW resonant frequency.

### 3D simulations of the microinductor on the giCPW board

Full-wave electromagnetic simulations were performed to assess the impact of dielectric losses up to 20 GHz on the complete measurement setup, comprising the gi-CPW board and the microinductor (Fig. S13). The simulations were based on the MoM (Feko Suite, Altair, USA). The lossy dielectric substrate (FR4) was modeled using a frequency-independent complex permittivity with *ε*_r_ = 4.3 and tan δ = 0.025, since the actual dispersive behavior of FR4 depends on the specific manufacturing batch and is generally not characterized by PCB suppliers. Under these assumptions, only dissipative effects could be reliably modeled.

Compared with the FEM, the MoM approach offers the advantage of meshing only the conductive geometries (CPW and microinductors), without requiring a large surrounding air volume. This enables an efficient multiscale meshing strategy, with the microinductor discretized using 10 µm local elements and the remaining structures meshed with a standard *λ*/10 resolution (Fig. S13). Under these conditions, the computation of S-parameters at the two CPW ports was performed on a workstation equipped with 64 GB RAM and a 4.1 GHz CPU, requiring approximately 24 h per simulation.

To replicate the experimental de-embedding procedure, a Transmission–Reflection–Line (TRL) calibration was implemented to isolate the DUT contribution^30,31^. This method is conceptually equivalent to the Auto Fixture Removal (AFR) routine available in Keysight VNAs, whose algorithm is proprietary and therefore not directly accessible. Specifically, three preliminary simulations of the PCB board were performed without the DUT, with the giCPW gap configured as: (i) short-circuited (Transmission), (ii) open (Reflection), and (iii) short-circuited with an additional *λ*/4 line section (Line, evaluated at 11 GHz, the center of the band). Subsequently, three additional simulations were carried out, including the microinductors with radii of 20, 40, and 60 μm bridging the giCPW gap, reproducing the full experimental configuration (Fig. S13).

Finally, an additional model was generated by replacing the FR4 substrate with a low-loss Rogers laminate (RO4350B) characterized by *ε*_r_ = 3.48 and tan δ = 0.0037. As shown in Fig. S13, the reduced dielectric losses yield a noticeable increase in *Q*-factor compared with FR4. Moreover, the excellent frequency stability of Rogers laminates up to several tens of gigahertz makes them promising candidates for future experimental prototypes.

## Supplementary information

Supplementary Information

Transparent Peer Review File

## Data Availability

The data that support the plots within this paper and other findings of this study are available from the corresponding author upon request.
