# Supplementary material for: On-chip 3D printing of metal microinductors for radio frequency electronics
Source: Nat Commun. 2026 Sep 8;17:10644. doi: 10.1038/s41467-026-77493-1 (PMC13649781; doi:10.1038/s41467-026-77493-1)
Supplement: Supplementary file 1 [file 41467_2026_77493_MOESM1_ESM.pdf]

Supporting Information

On-Chip 3D Printing of Metal Microinductors for Radio Frequency Electronics

Alessandro Paghi[1,2+], Farbod Abazar[1+], Valeria Lazzoni[1], Angelica Masi[1], Sabrina Rotundo[1], Danilo Brizi[1], Agostino Monorchio[1], and Giuseppe Barillaro[1,3*]

[1]Dipartimento di Ingegneria dell'Informazione, Università di Pisa, via G. Caruso 16, 56122 Pisa, Italy.

[2]NEST, Istituto Nanoscienze-CNR and Scuola Normale Superiore, Piazza S. Silvestro 12, 56127 Pisa, Italy.

[3]Centro per l'Integrazione della Strumentazione dell'Università di Pisa (CISUP), Lungarno Pacinotti 43, Pisa I-56126, Italy

[+] These authors contribute equally to this work

[*]corresponding author: giuseppe.barillaro@unipi.it

Table S1 Comparison of air-core microinductors fabricated using different technologies

Fabrication technology	Geometry	Inductance	Quality factor	Self-resonance frequency	Maximum handling current	Footprint area on chip	Compatibility with on-chip integration	Application	Year	Ref
3D printing	Solenoid	0.8 nH (r=20 μm)<br>1.8 nH (r=40 μm)<br>3 nH (r=60 μm)	17 (r=20 μm) at 5GHz<br>18 (r=40 μm) at 5GHz<br>18 (r=60 μm) at 5GHz	50-250 GHz	200 mA	0 [a]	YES	RF	2026	This work
	Solenoid	6.67nH at 24.79GHz [b]	NA	25.4 GHz	NA	30000 μm²	YES	RF	2019	1
Self-rolled up assembly	3D spiral	1 to 5nH at 5GHz	17 at 12GHz	40 to 53 GHz	NA	80000 μm² (pre-roll)	YES	RF	2025	2
	3D spiral	140nH at 10MHz	2.85 at 250MHz	2 GHz	250 mA	1.4 mm² (pre-roll)	NO	RF	2020	3
TSV fabrication process	Solenoid	86.6nH at 51MHz	37.6 at 21MHz	NA	NA	100000 μm²	YES	Power	2019	4
	Solenoid, Toroidal	34.2-44.6nH at 100MHz	10–13 at 30–72MHz	NA	NA	1.76 mm²	YES	Power	2018	5
	Spiral	13.1nH at 100MHz	3.9 at 100MHz	NA	2 A	630000 μm²	YES	Power	2011	6
Wire-bonding	Solenoid	600nH at 400MHz	52 at 400MHz	2.6 GHz	NA	780000 μm²	YES	RF	2010	7
3D surface micromachining	Solenoid	0.45nH at 33GHz	25 at 33GHz	> 40GHz	NA	60000 μm²	YES	RF	2018	8
	Toroidal	210nH at 30MHz	12 at 6MHz	> 20MHz	NA	78.53 mm²	NO	Power	2013	9
	Solenoid	2.96nH at 5.35GHz	45 at 5.35GHz	10 GHz	NA	120000 μm²	YES	RF	2007	10
	Solenoid	2.2 to 3.6nH	23.7 at 4.5GHz	NA	NA	702 μm²	YES	RF	2002	11
2D surface micromachining	Spiral	36-420nH at 10-100MHz	16 at 50MHz	NA	NA	7.29 mm²	YES	Power	2011	12
	Spiral	4.2nH at 2GHz	37 at 2GHz	8.7 GHz	NA	250000 μm²	YES	RF	2004	13
	Spiral	5nH at 2.4GHz	15 at 2.4GHz	10 GHz	NA	30000 μm²	YES	RF	2003	14

a Solenoid printed on contact pads,
b Theoretical calculated value

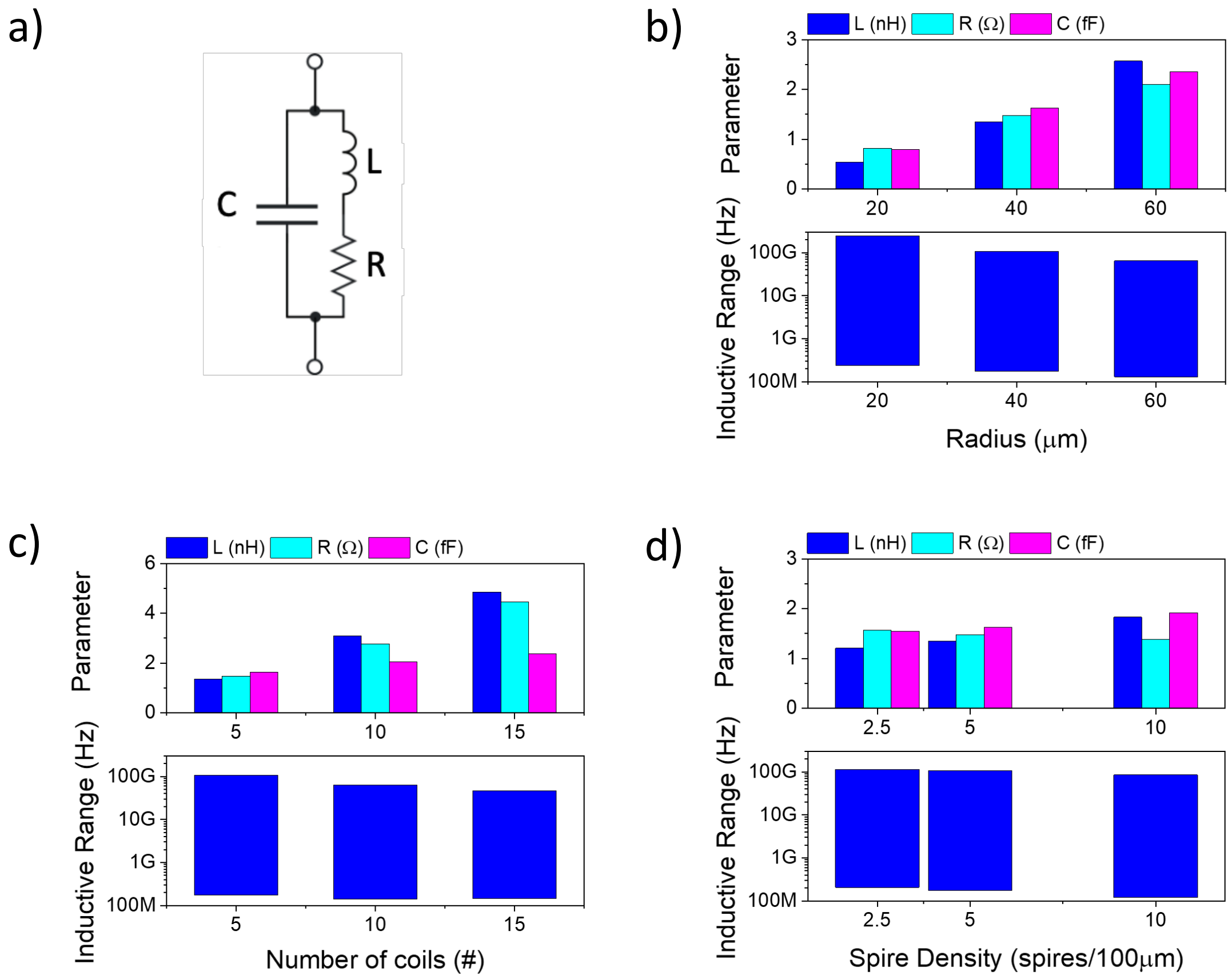

Figure S1. Lumped electrical model parameters of the printed Cu microinductors extracted from 3D simulations. a) Lumped element equivalent circuit of the printed Cu microinductors up to 2 GHz. b,c,d) Lumped element values (top) and inductive range (bottom) of microinductors featuring Cu solenoids with turn of different radii (b), number (c), and density (d) up to 2 GHz.

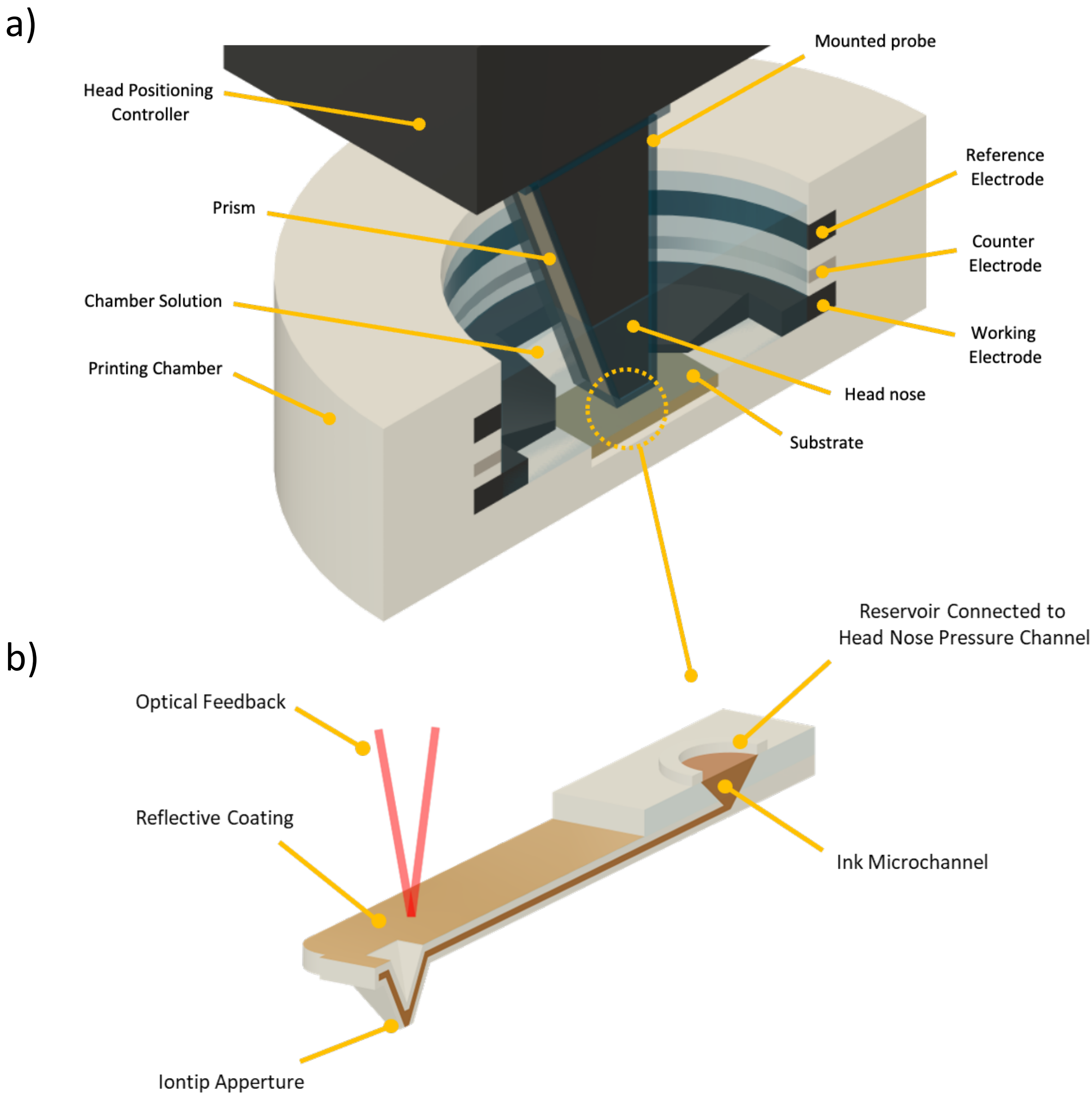

Figure S2. 3D sketch of the EXADDON CERES printing system. a) A 3D sketch of the printing head inside the printing chamber. The printing head, which mounts the iontip, is submerged in the chamber solution (i.e. supporting electrolyte) housed in the three-electrode printing chamber. b) A 3D sketch of the iontip. The iontip features a nozzle with an opening of 300 nm connected with a microchannel integrated within the iontip. The printing ink, containing metal ions dissolved in an electrolyte solution, is propelled through the tip from a reservoir at the other end of the microchannel.

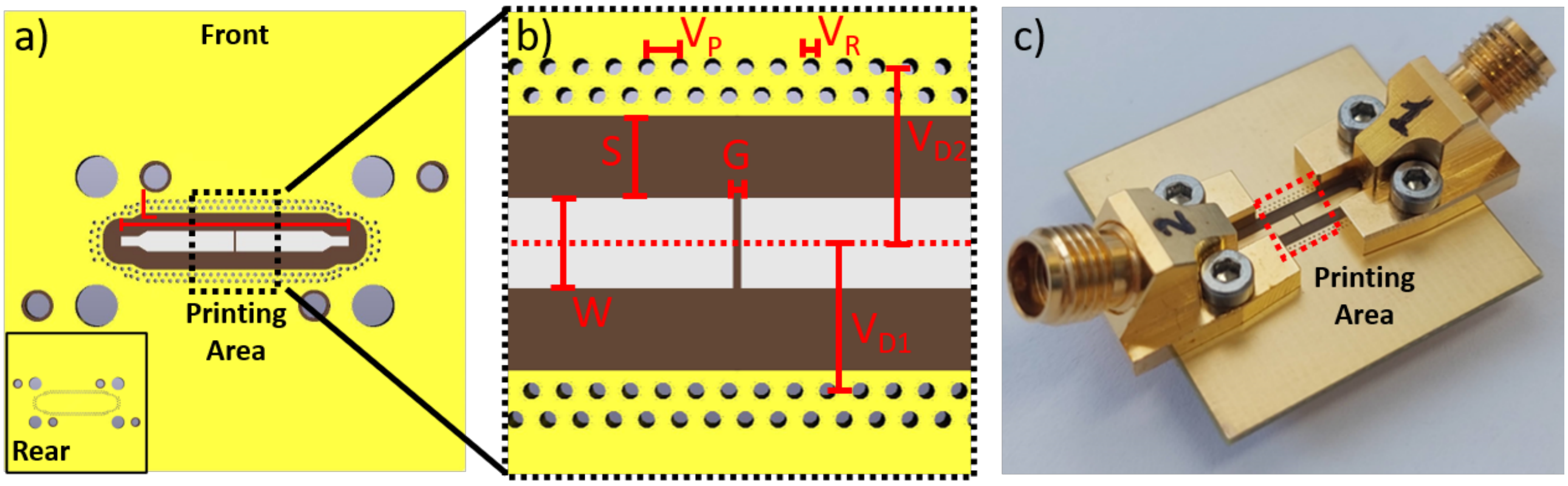

Figure S3. Gap-interrupted grounded coplanar waveguide (giCPW) printed circuit board (PCB). a) CAD top view of the PCB exhibiting the giCPW layout. The inset at the bottom-left corner shows the rear view. b) CAD top-view magnification of the area of the giCPW devoted to microinductor printing. c) Picture of the fabricated giCPW PCB provided with two solderless PCB mount SMA connectors.

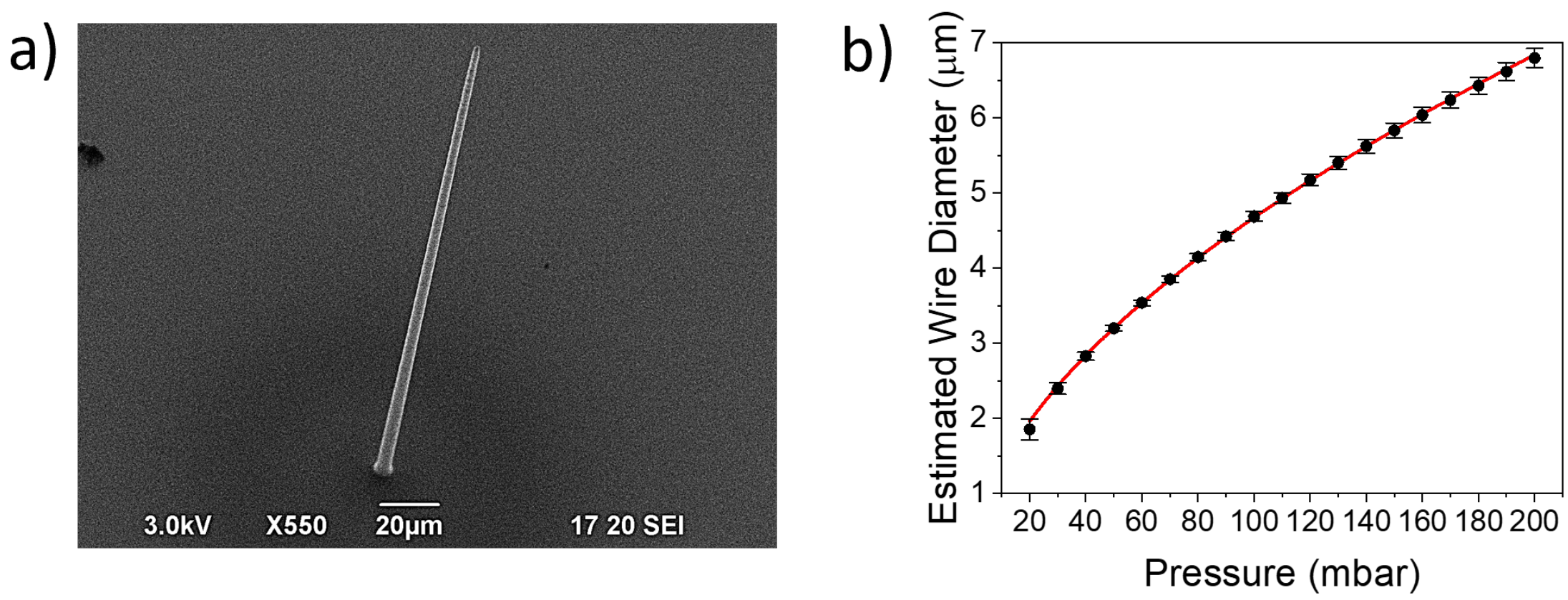

Figure S4. Characterization of the iontip printing performance. a) Tilted-view SEM image of a Cu calibration pillar (height = 150 µm), printed with extrusion pressures from 20 to 200 mbar onto a gold substrate; the scalebar is 20 µm. b) Calibration curve showing the estimated wire diameter versus extrusion pressure, obtained from the 3D-printed calibration pillar shown in (a) (n=3 samples). Data in b) are reported as mean value ± error bar representing the standard deviation.

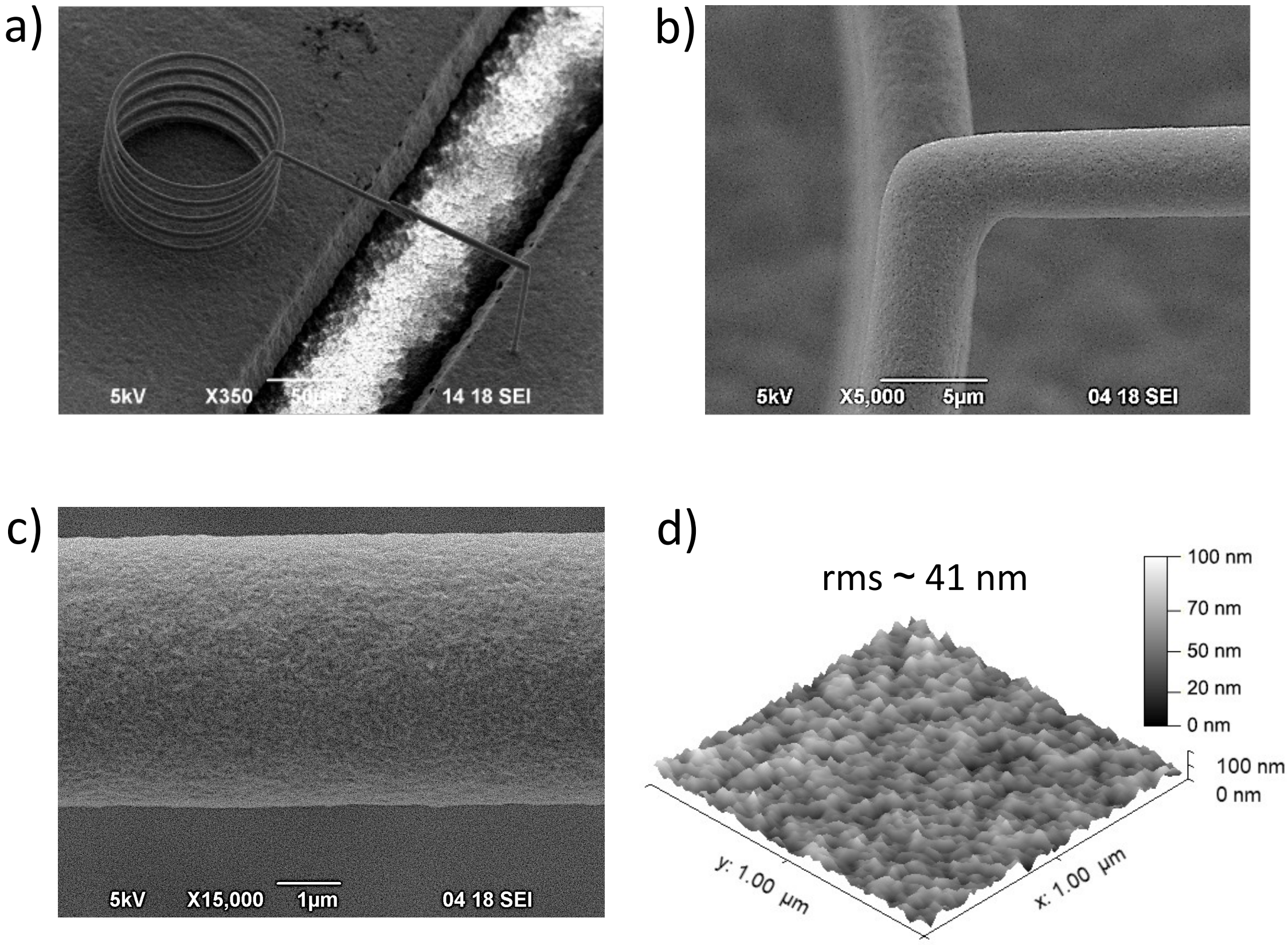

Figure S5. Morphological characterization of on-chip 3D printed metal microinductors. a) Tilted-view SEM image of a microinductor featuring a Cu microsolenoid with 5 turns (radius = 60 µm, wire diameter = 5 µm, turn density = 5 spires/100µm), printed onto a giCPW; the scalebar is 50 µm. b,c) SEM images of a 90 ° bend (b) and horizontal wire (c) of the microinductor in a) ; the scalebars are 5 µm (b) and 1 µm (c). d) Grayscale roughness profile typical of the printed microindcutor obtained over a 1×1 µm surface area in (c).

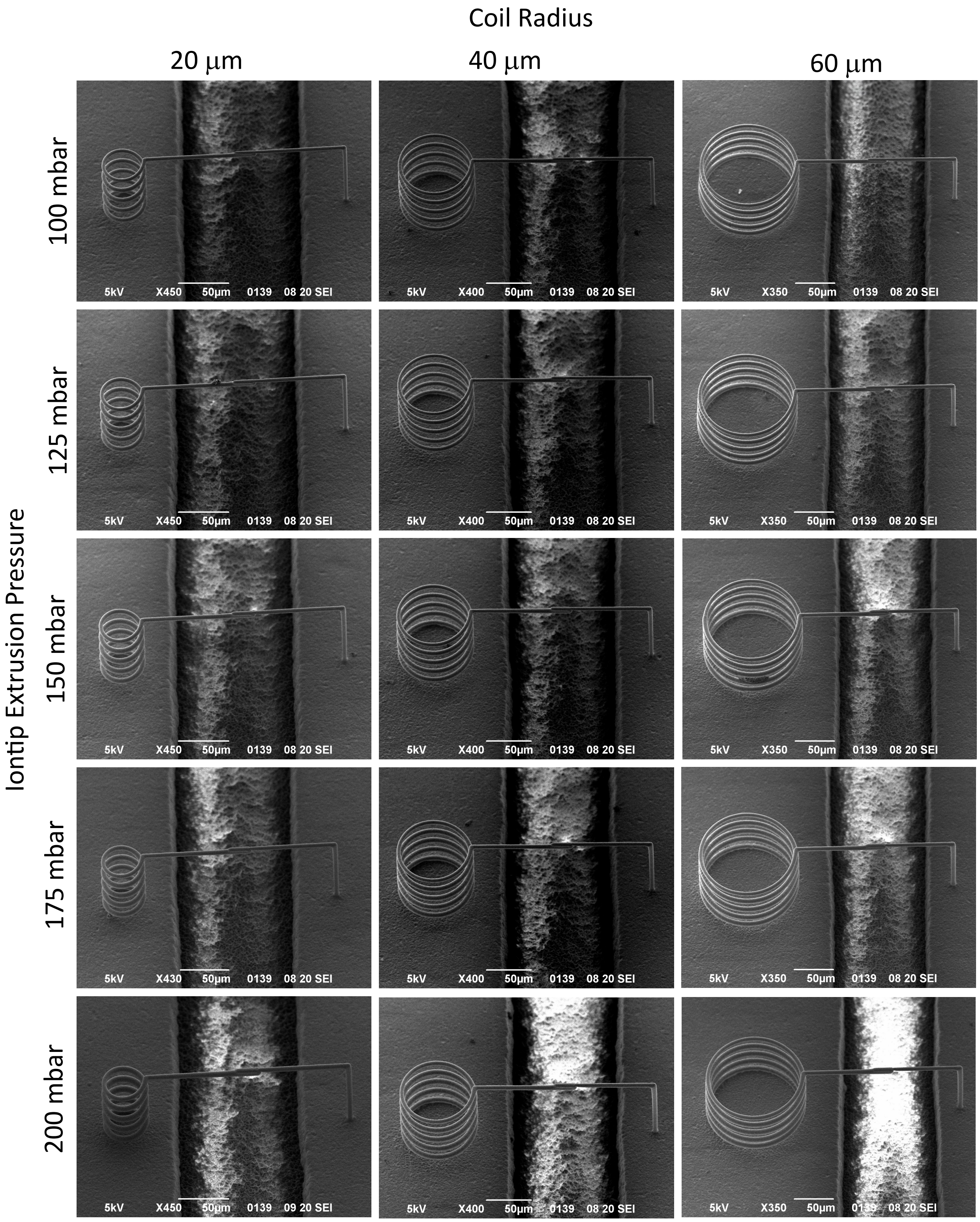

Figure S6. Tilted-view SEM images of microinductors featuring turn with different radii fabricated with various iontip extrusion pressures. The microinductors with turn radius of 20, 40 and 60 μm, 5 turns, and turn density of 5 turns/100μm were printed on the giCPW with various extrusion pressures of 100,125,150,175 and 200 mbar.

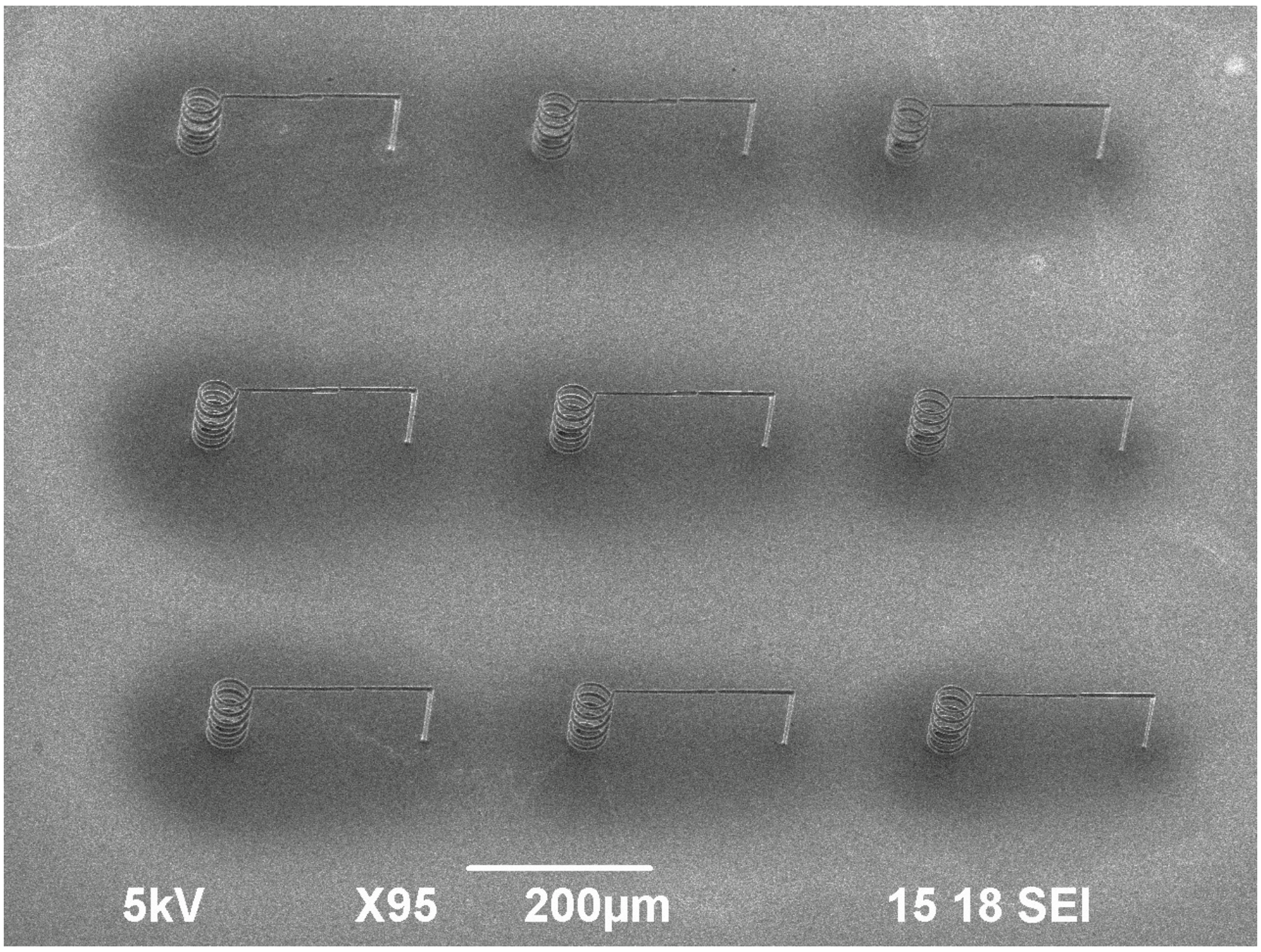

Figure S7. Tilted-view SEM images of an array of printed microinductors. Array of Cu microinductors with radius of 20 µm, 5 turns, and density of 5 turns/100µm, printed with an extrusion pressure of 200 mbar.

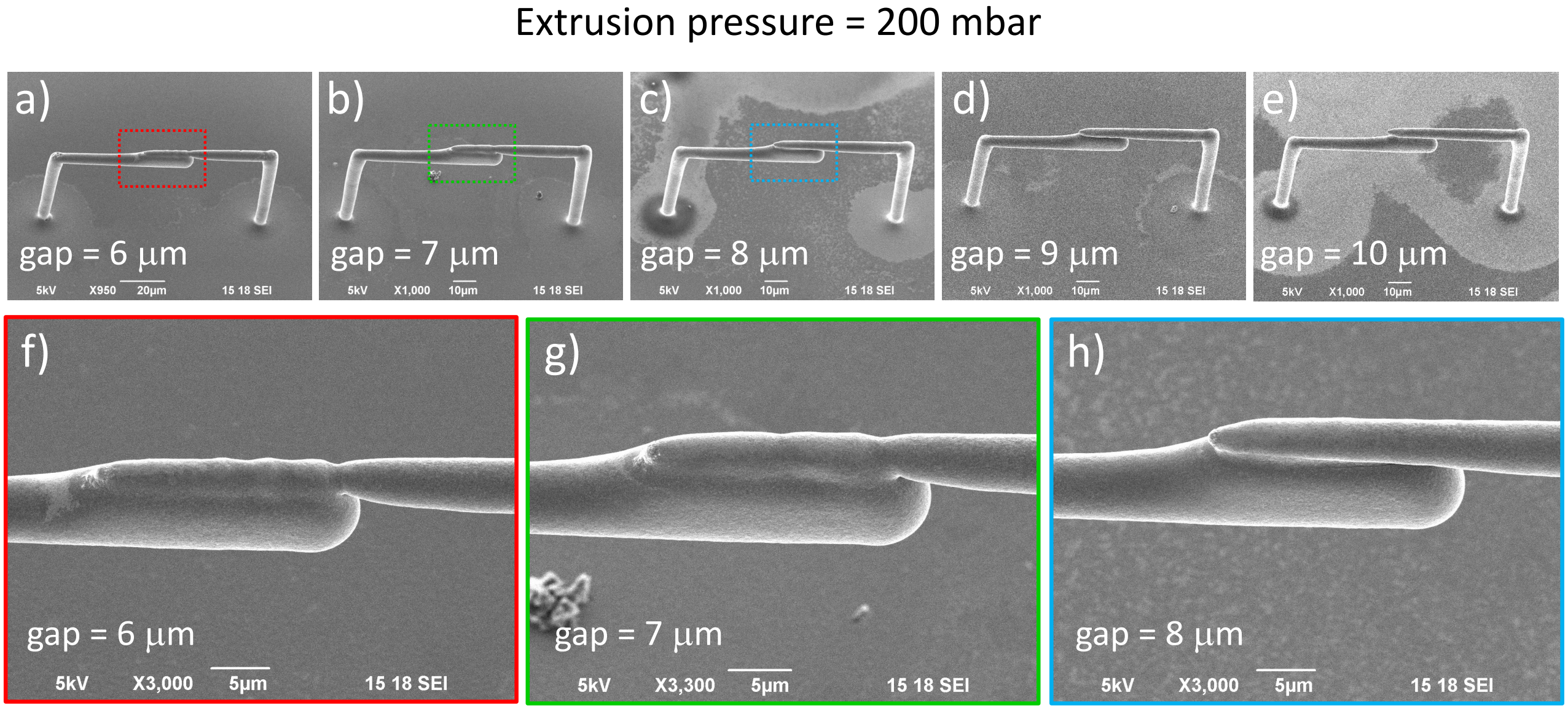

Figure S8. Tilted-view SEM images of test structures for optimizating the electrical connection. All the structures were printed with an extrusion pressure of 200 mbar, and different vertical gaps from 6 to 10 µm between the horizontal wires: a,f) gap 6 µm; b,g) gap 7 µm; c,h) gap 8 µm; d) gap 9 µm; e) gap 10 µm.

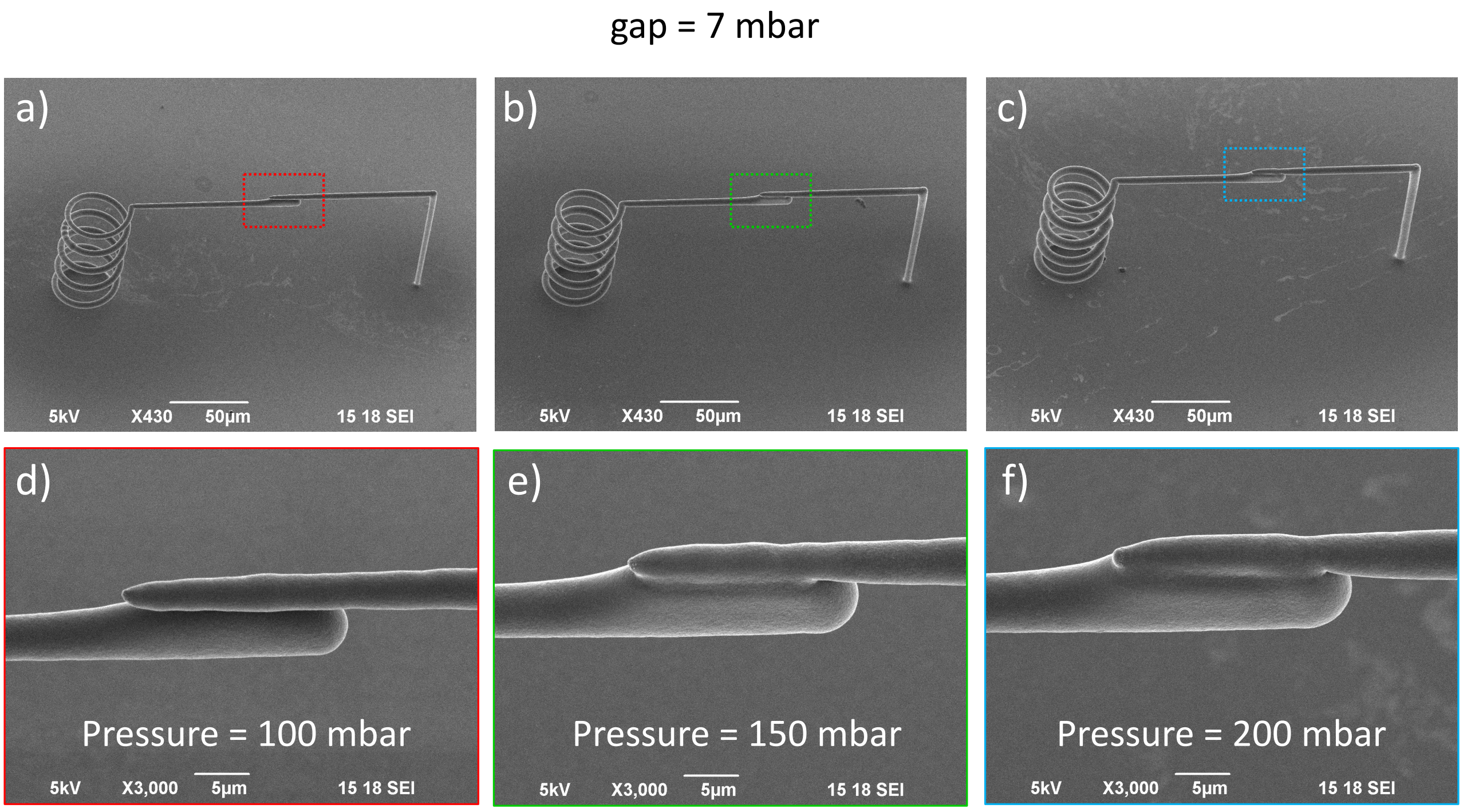

Figure S9. Tilted-view SEM images of microinductors for optimizing the electrical connection. All the Cu microsolenoids were printed with a gap of 7 µm between the two horizontal wires, at different extrusion pressures from 100 to 200 mbar: a,d) pressure 100 mbar; b,e) pressure 150 mbar; c,f) pressure 200 mbar.

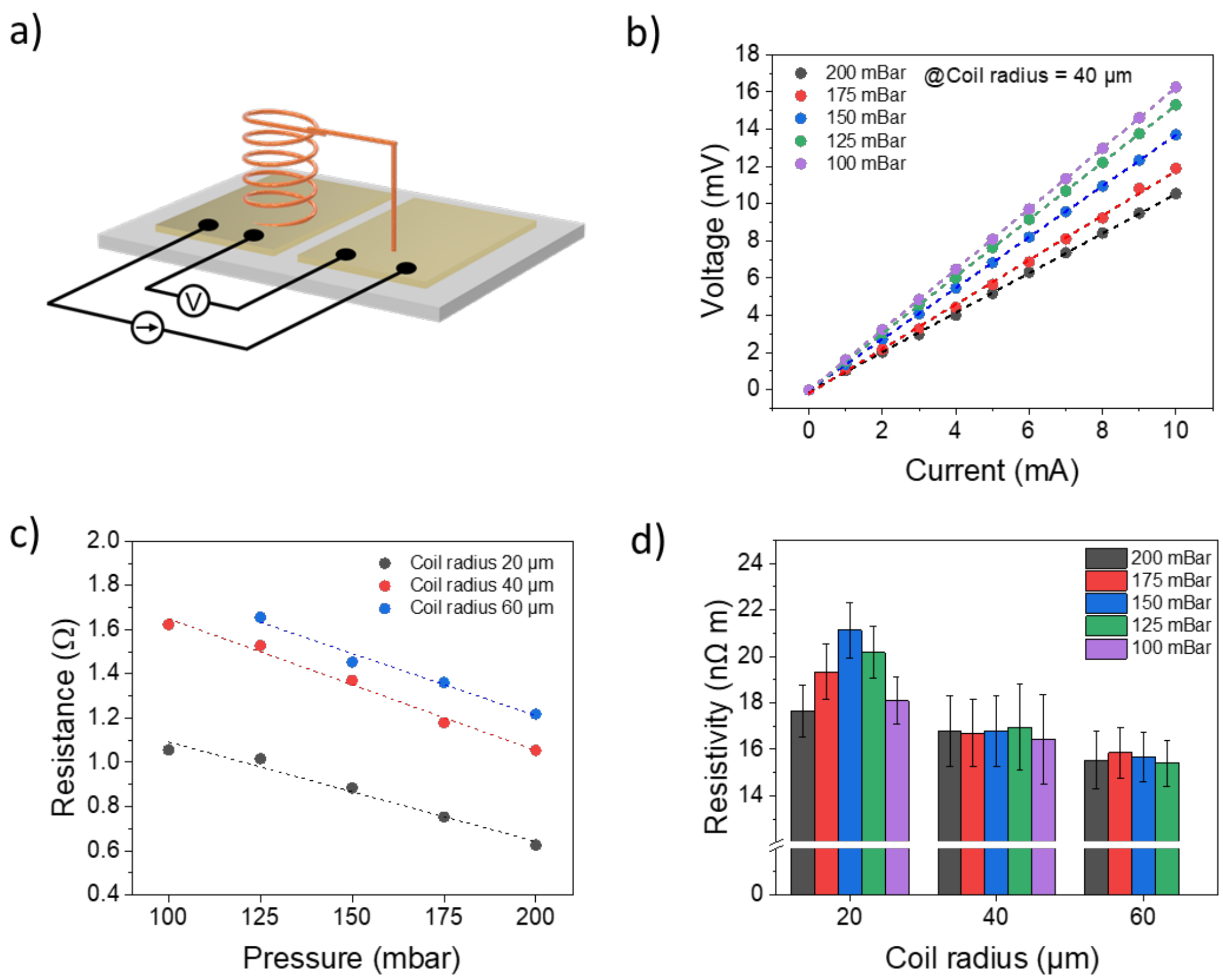

Figure S10. DC Electrical characterization of on-chip 3D printed metal microinductors. a) Sketch of the 4-probes electrical measurement configuration used to measure the DC resistance of microinductors printed on a giCPW PCB. b) Voltage vs. current curves of microinductors featuring a Cu solenoid with 5 turns (radius = 40 µm, density = 5 turns/100µm), printed on a giCPW at different extrusion pressures. c) DC resistance vs. extrusion pressure of microinductors featuring Cu solenoids with 5 turns (density = 5 turns/100µm) and different radii, printed onto a giCPW. d) DC resistivity values of copper printed at different extrusion pressures for microsolenoids with varying radii (n=3 samples for each experimental conditions). Data in d) are reported as mean value ± error bar representing the standard deviation.

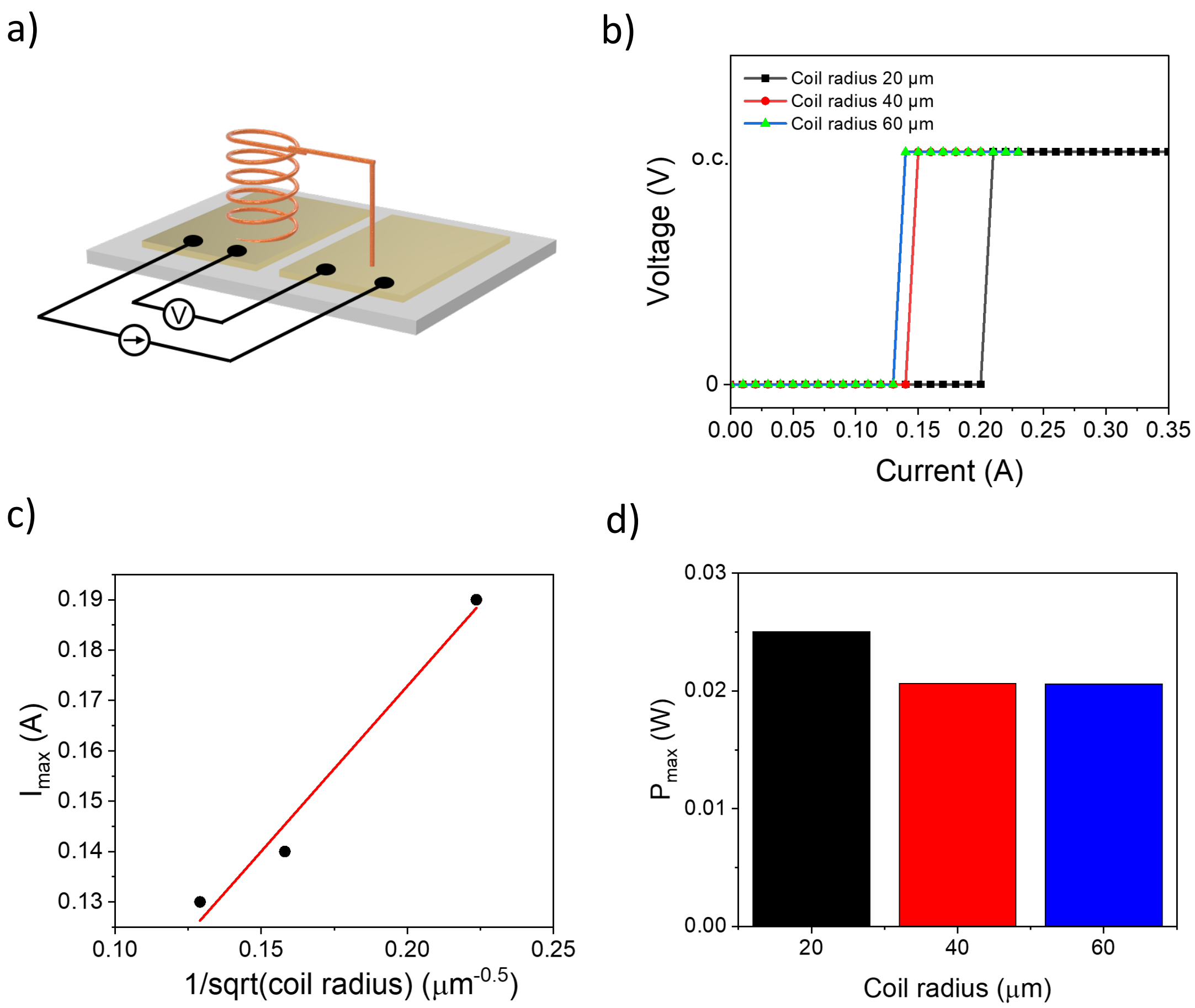

Figure S11. Maximum current and power of printed Cu microinductors. a) Sketch of the 4-probes electrical measurement configuration used to measure the DC resistance of microinductors printed on a giCPW PCB. b) Voltage vs. current curves of microinductors featuring solenoids with 5 turns (density = 5 turns/100μm) and different radii, printed onto a giCPW. "o.c." means that the SMU reached the compliance voltage due to the destruction of the microinductor at $I = I_{max}$. c) Maximum microinductor current vs. 1/√radius. The red line is the theoretical best-fitting curve to the experimental data. d) Maximum power vs. solenoid radius extrapolated as $P_{max} = R \times I_{max}^2$.

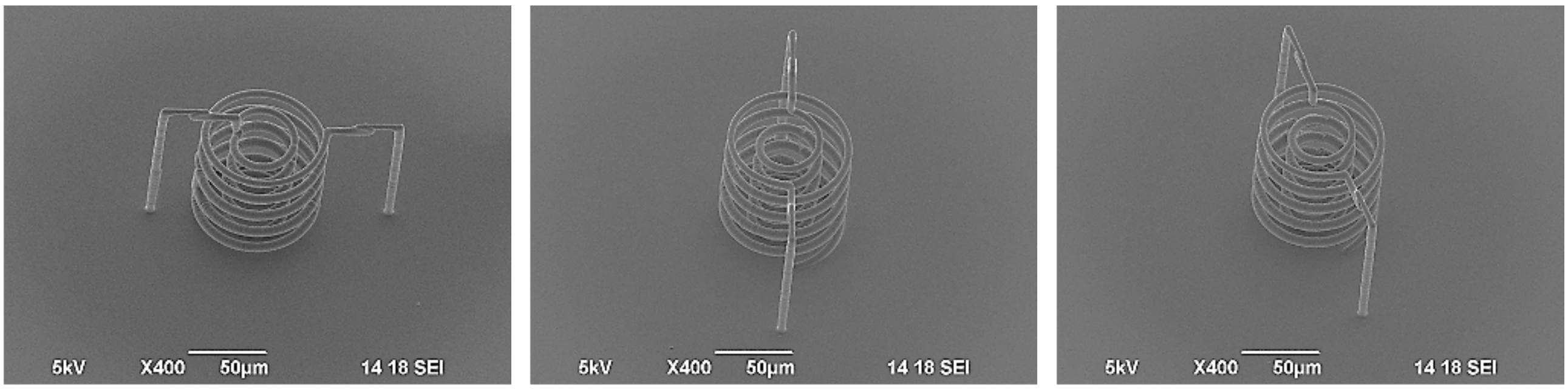

Figure S12. Tilted-view SEM images of two concentric microinductors printed in transformer configuration. The microinductors feature turns with a radius of 20 µm (inner) and 40 µm (outer), 5 turns, and density of 5 turns/100µm.

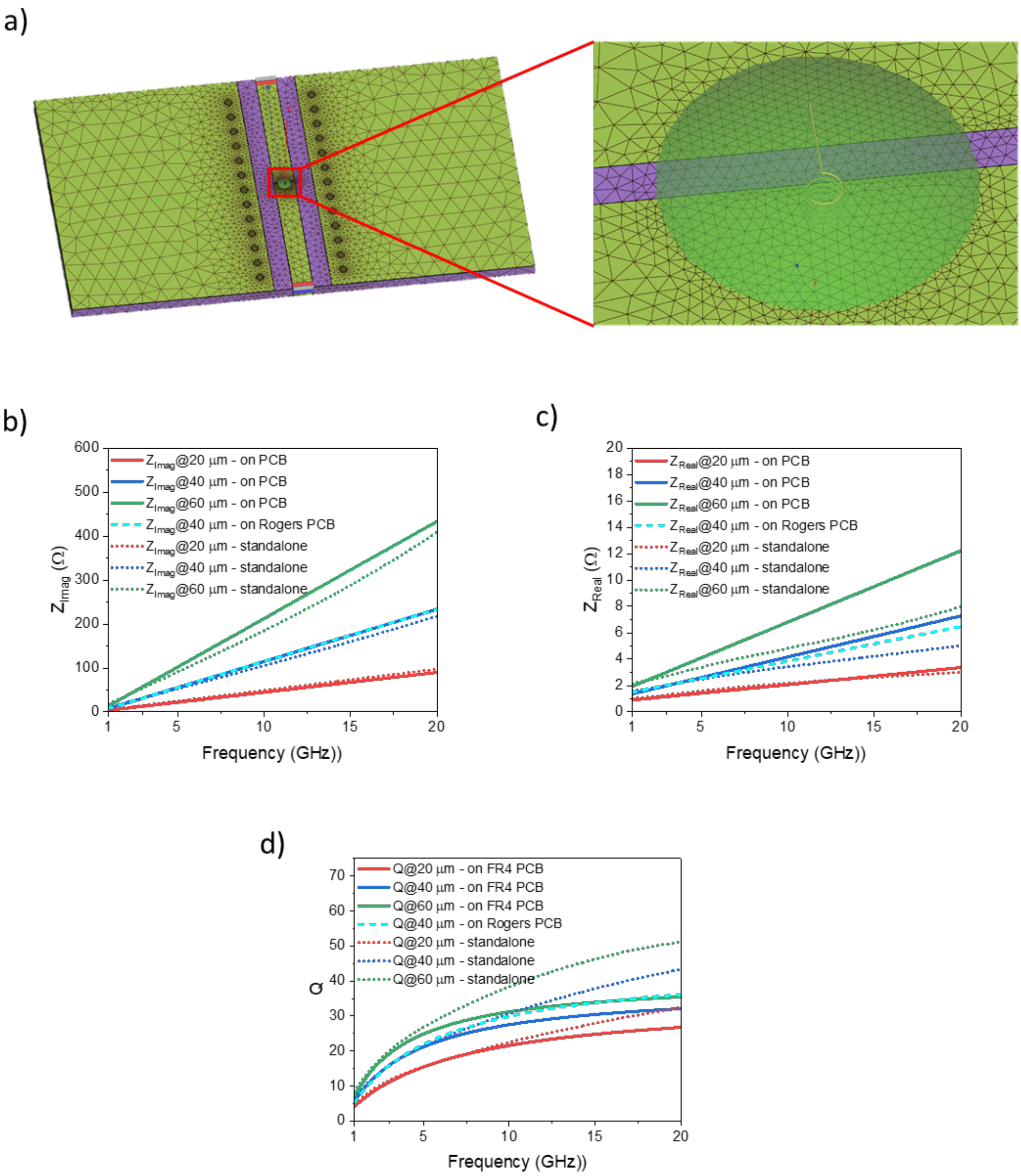

Figure S13. Electromagnetic simulations of microinductors on FR4 substrates. (a) Simulated geometry of a representative microinductor printed across the coplanar waveguide (CPW) gap, with a zoom-in on the solenoid region. (b) Imaginary part of the impedance as a function of frequency for microinductors with different radii, comparing micronductors standalone and printed on FR4. (c) Real part of the impedance for the same inductors, highlighting the deviation introduced by substrate losses. (d) Frequency dependence of the Q-factor, showing saturation for FR4-supported inductors beyond a few gigahertz, in contrast with the steady increase observed for standalone devices.

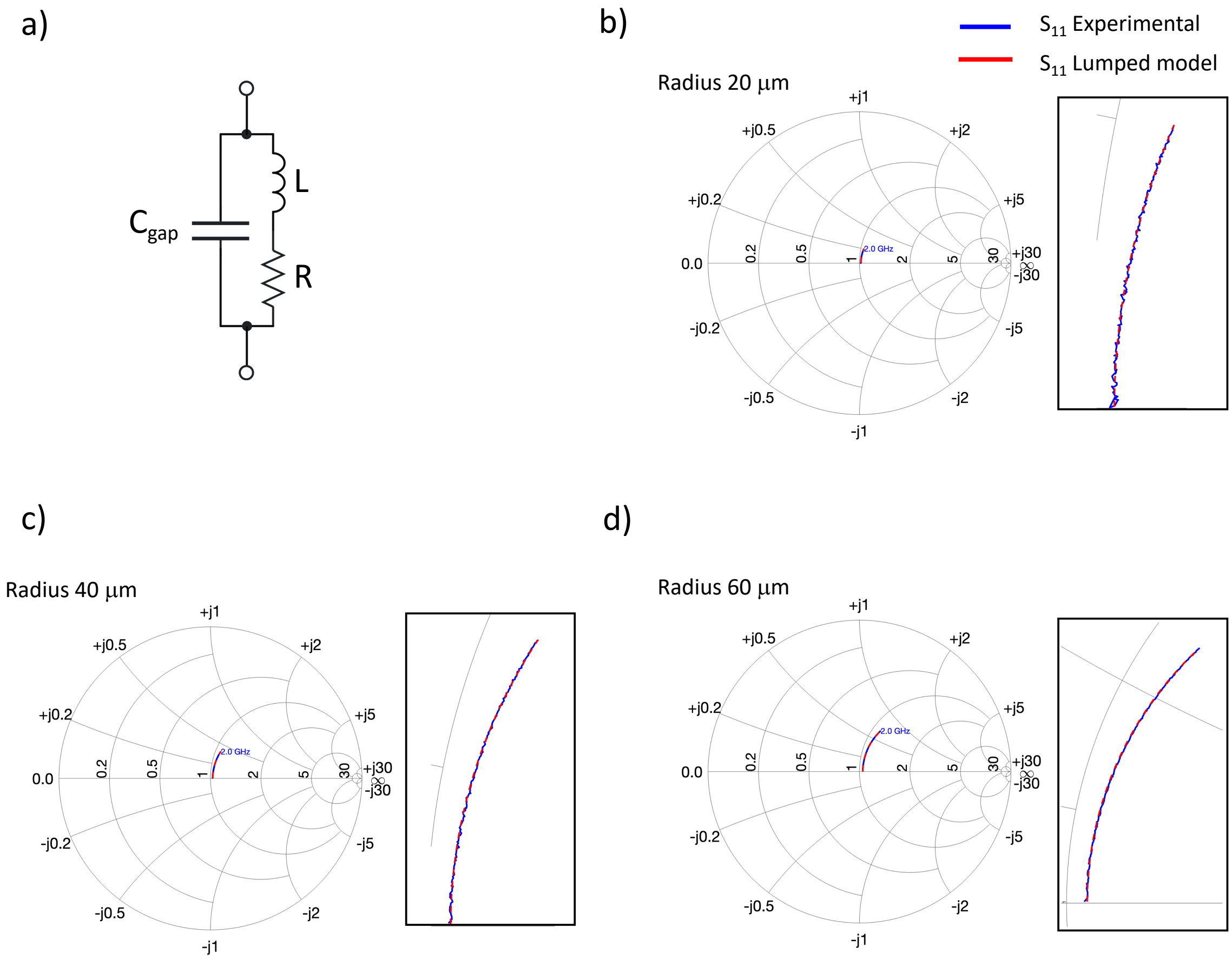

Figure S14. Validation of lumped-element modeling for 3D-printed microsolenoids. a) Schematic representation of the lumped-element equivalent circuit used to model the printed microsolenoids, consisting of a series inductance (L) and resistance (R), and a parallel parasitic capacitance (C_{gap}) representing the intrinsic capacitance of the coplanar waveguide (CPW). b–d) Smith chart representations of the measured two-port S_{11} parameters (solid blue) and the fitted lumped-element model (dashed red) for microsolenoids with radii of 20 µm (b), 40 µm (c), and 60 µm (d), respectively. Insets show a magnified view of the high-frequency region up to 2 GHz. The close agreement between experiment and model across the entire frequency range confirms the accuracy of the equivalent circuit representation.

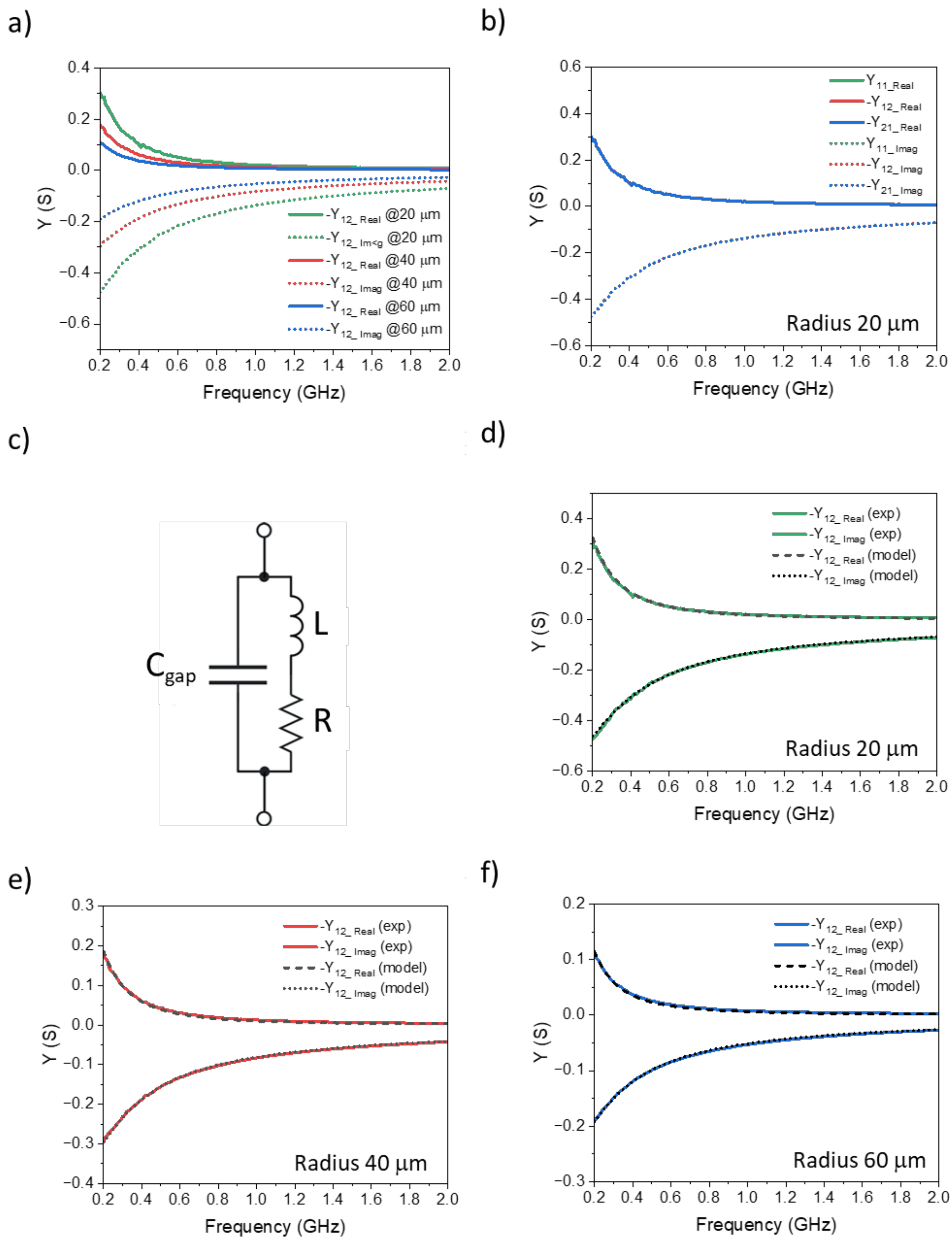

Figure S15. Validation of printed microinductors using Y-parameters. (a) Real and imaginary parts of the Y_{12} parameter, extracted from S-parameter measurements, for microinductors with different radii. (b) Comparison of Y_{11}, Y_{12}, and Y_{21} for a microinductor with radius of 20 µm, confirming the expected relation $Y_{11} = -Y_{12} = -Y_{21}$. (c) Equivalent lumped-element model of the microinductor, consisting of a series resistance R and inductance L in parallel with a parasitic capacitance C_p. (d-f) Best-fit curves of the lumped model overlaid with the measured Y-parameters of the devices in (a), showing excellent agreement between experiment and theory and yielding R and L values consistent with those extracted directly from S-parameters.

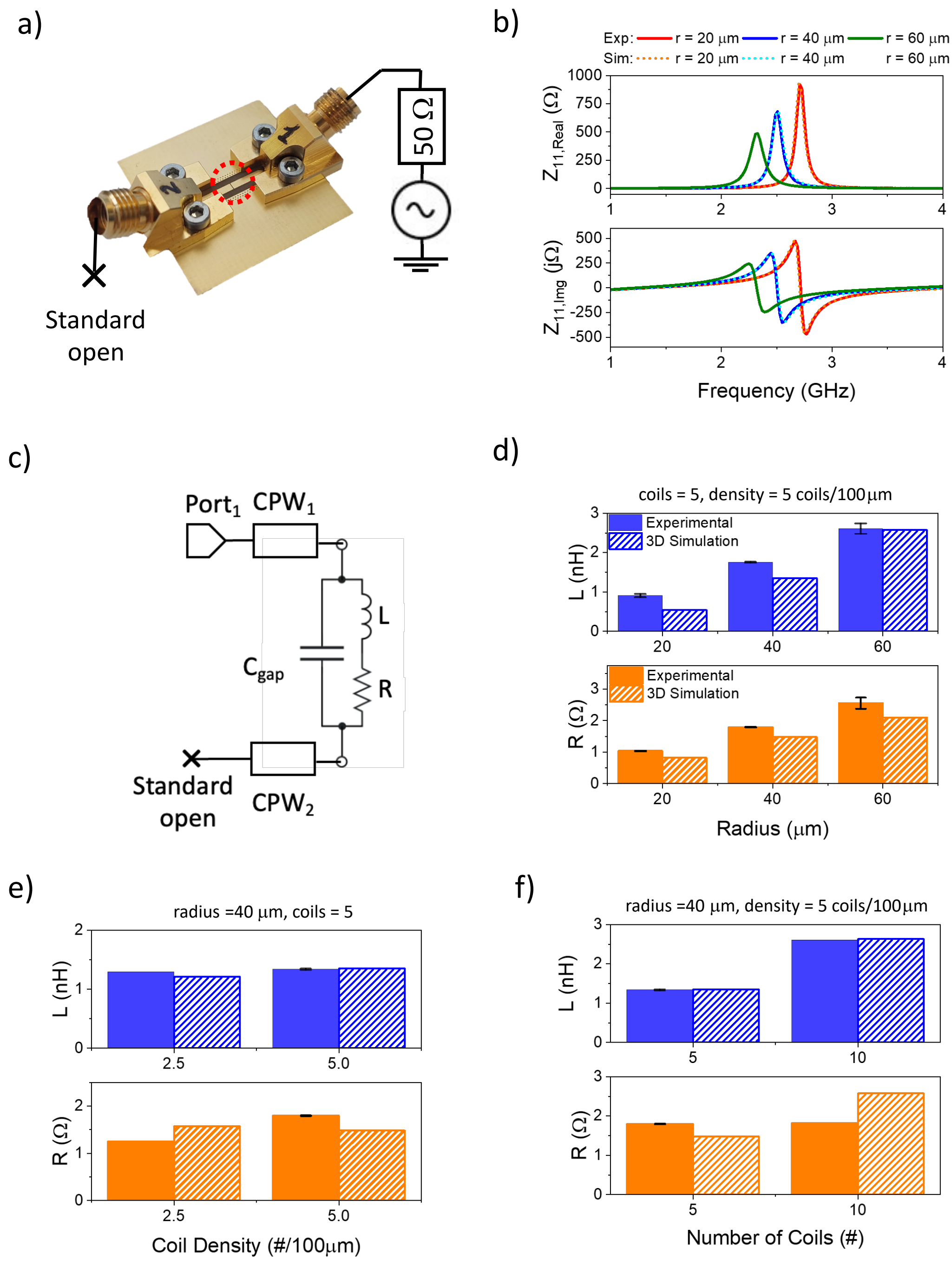

Figure S16. 1-port resonant measurements of 3D printed metal microinductors. a) Picture of the giCPW PCB highlighting the printing area with a red dashed circle. b) Experimental (solid traces) input impedance Z_{11} vs. frequency of the giCPW with one end left open, measured for different printed microinductors featuring solenoids with varying radii. Best-fitting theoretical curves to experimental data (dotted traces) achieved used the equivalent model in c) are also reported. c) Equivalent circuit of the giCPW with printed microinductors used to best-fit experimental frequency-resolved Z_{11} data. d-f) Experimental (n=3 per radius) and 3D simulation values of electrical lumped-model parameters (L and R) of microinductors featuring solenoids with different radii (d), turn densities (e), and number of turns (f) printed onto the giCPW.

Experimental data in d), e), f) are reported as mean value ± error bar representing the standard deviation.

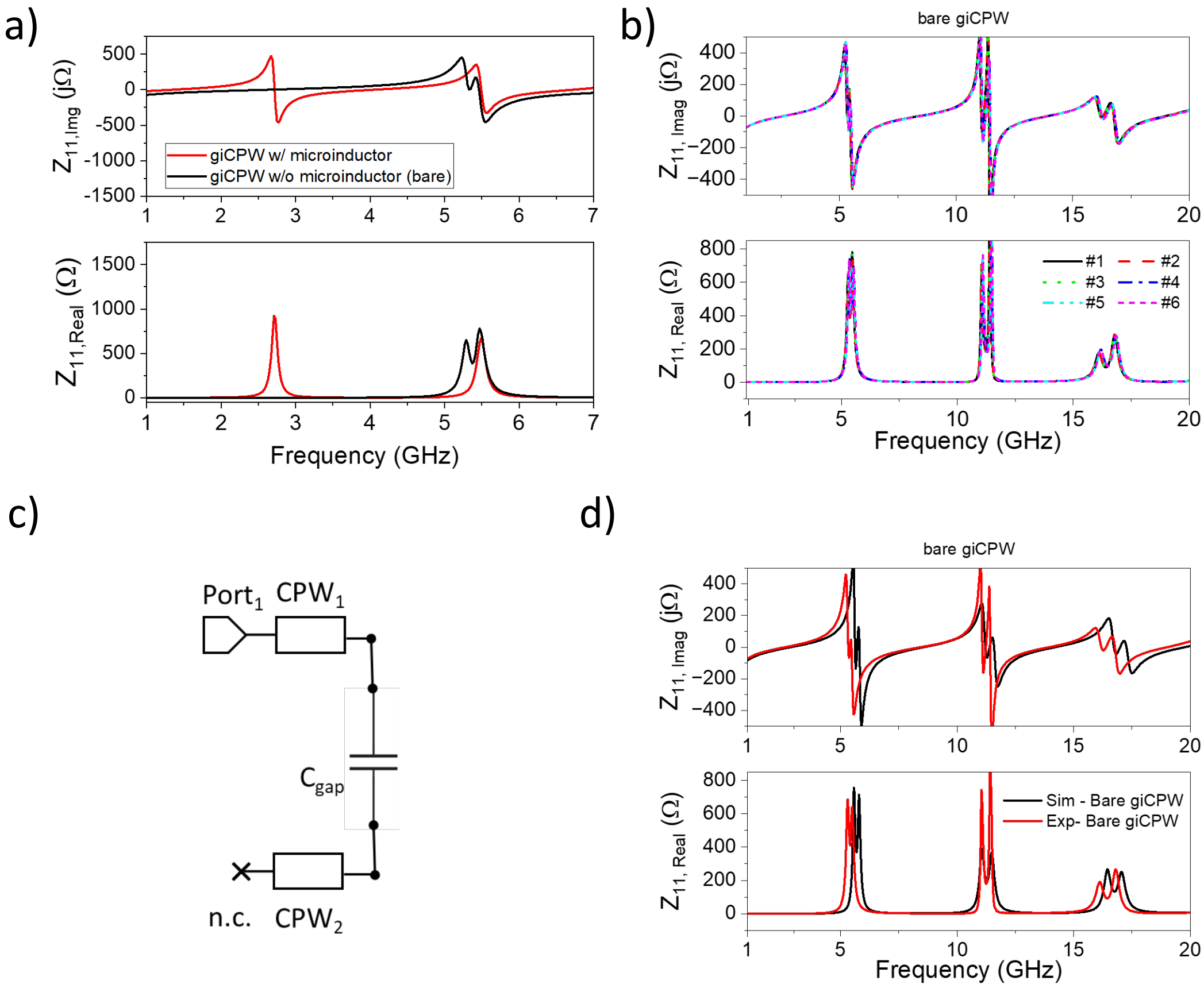

Figure S17. 1-port electrical characterization of giCPW before and after printing of microinductors. a) Experimental impedance vs. frequency diagrams of the giCPW PCB measured before (black trace) and after (red trace) printing of microinductors. b) Experimental impedance vs. frequency diagrams of six different giCPW PCBs, before printing of the microinductors. c) Lumped element equivalent circuit of a the giCPW. d) Experimental and simulated impedance vs. frequency diagrams of the giCPW before printing of the microinductors.

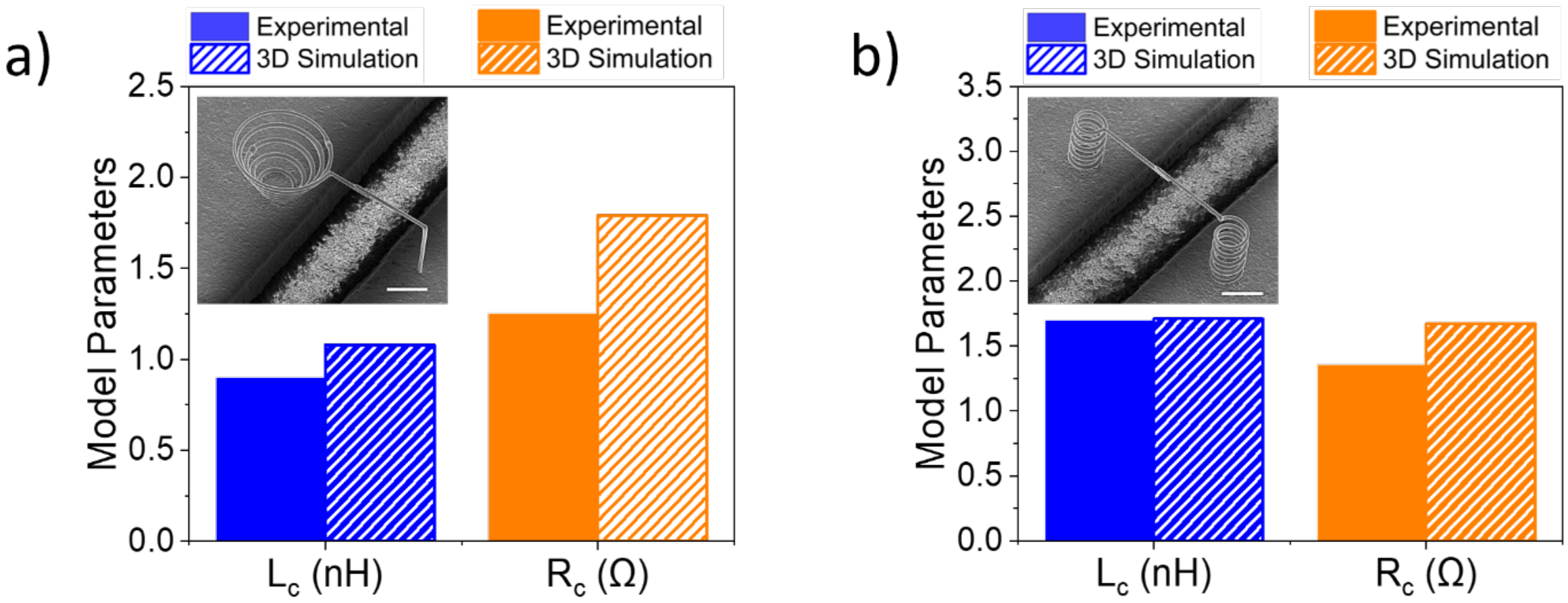

Figure S18. Electrical characterization of on-chip printed metal microinductors with turns of variable radius and connected in series. Experimental and 3D simulation values of inductance and resistance of microinductors featuring solenoids with turns of variable radius (a) and in series (b), printed onto a giCPW PCB; the inset SEM images show the printed structures; the scalebar is 50 μm.

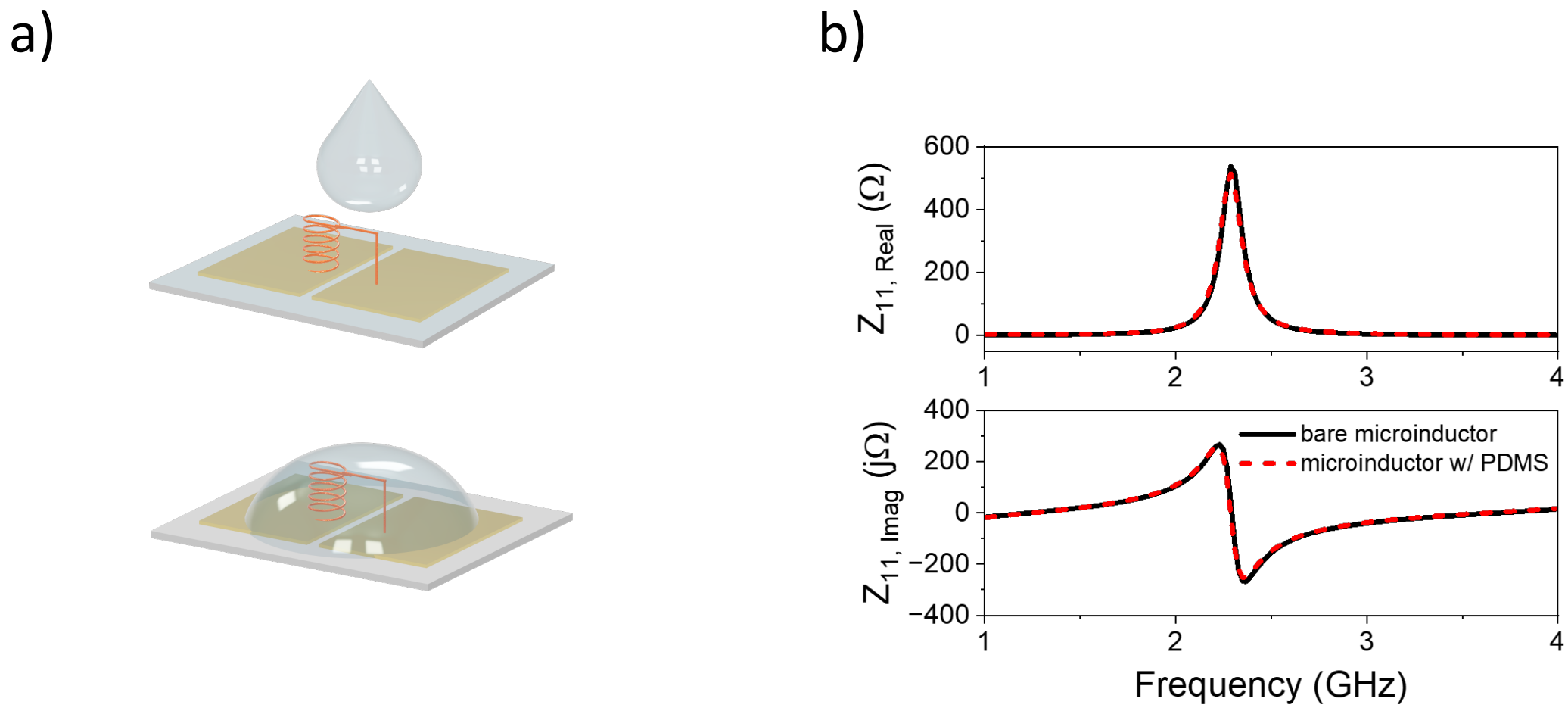

Figure S19. On-chip encapsulation of 3D printed microinductors with PDMS. a) Sketch of the microinductor encapsulation process: a volume of 10 µl of PDMS pre-polymer is dropped onto the printed microinductor (top) and then thermally cured at 60°C for 1 hour to crosslink the polymer (bottom). b) Experimental impedance vs. frequency of a microinductor featuring a solenoid with radius of 60 µm, 5 turns, and density of 5 turns per 100 µm, before (solid traces) and after (dotted traces) encapsulation in PDMS.

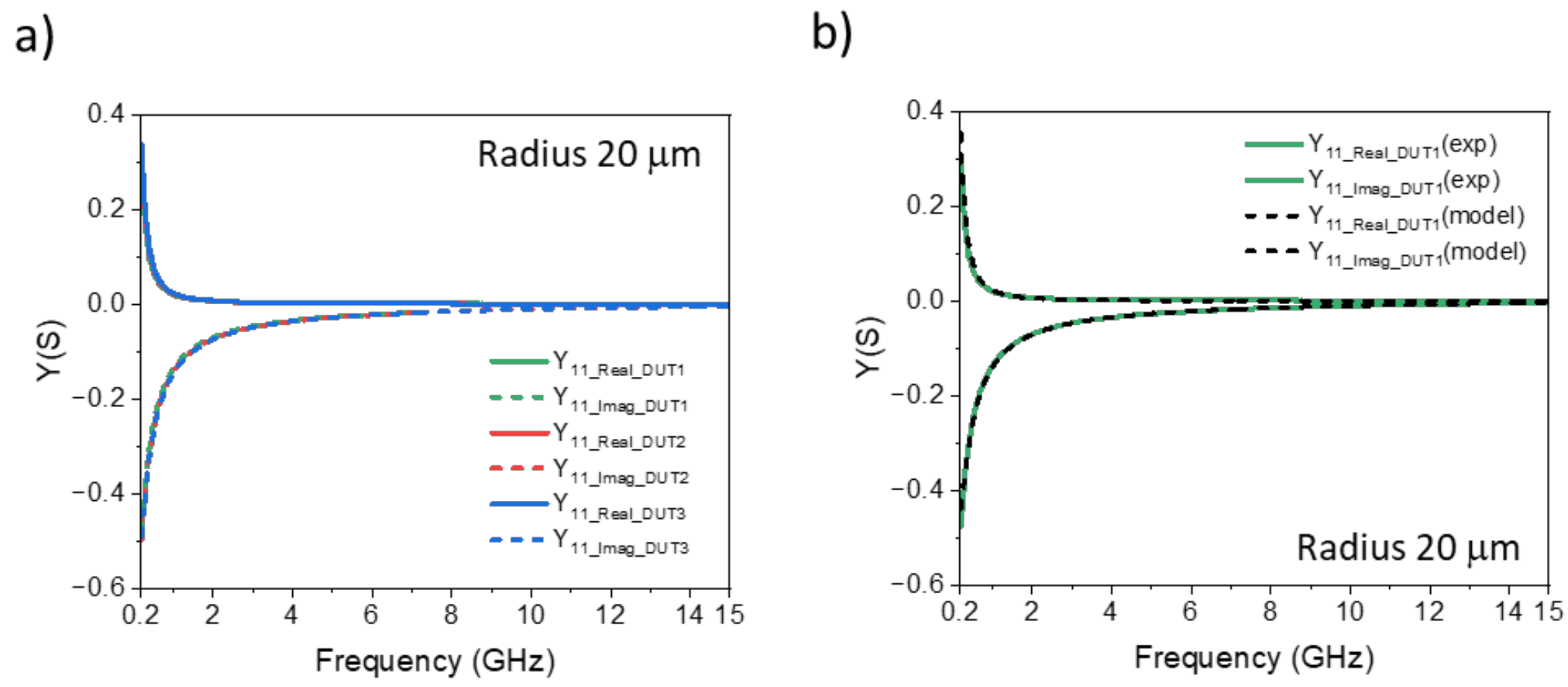

Figure S20. Validation of printed microinductors up to 15 GHz using Y-parameters. a) Real and imaginary components of the admittance parameters (Y) as a function of frequency for three microsolenoids with radius of 20 µm, extracted from S-parameter measurements up to 15 GHz. b) Real and imaginary components of the admittance for a representative microsolenoid from (a), showing experimental data (solid lines) and corresponding fits using a lumped-element RLC model (dotted lines).

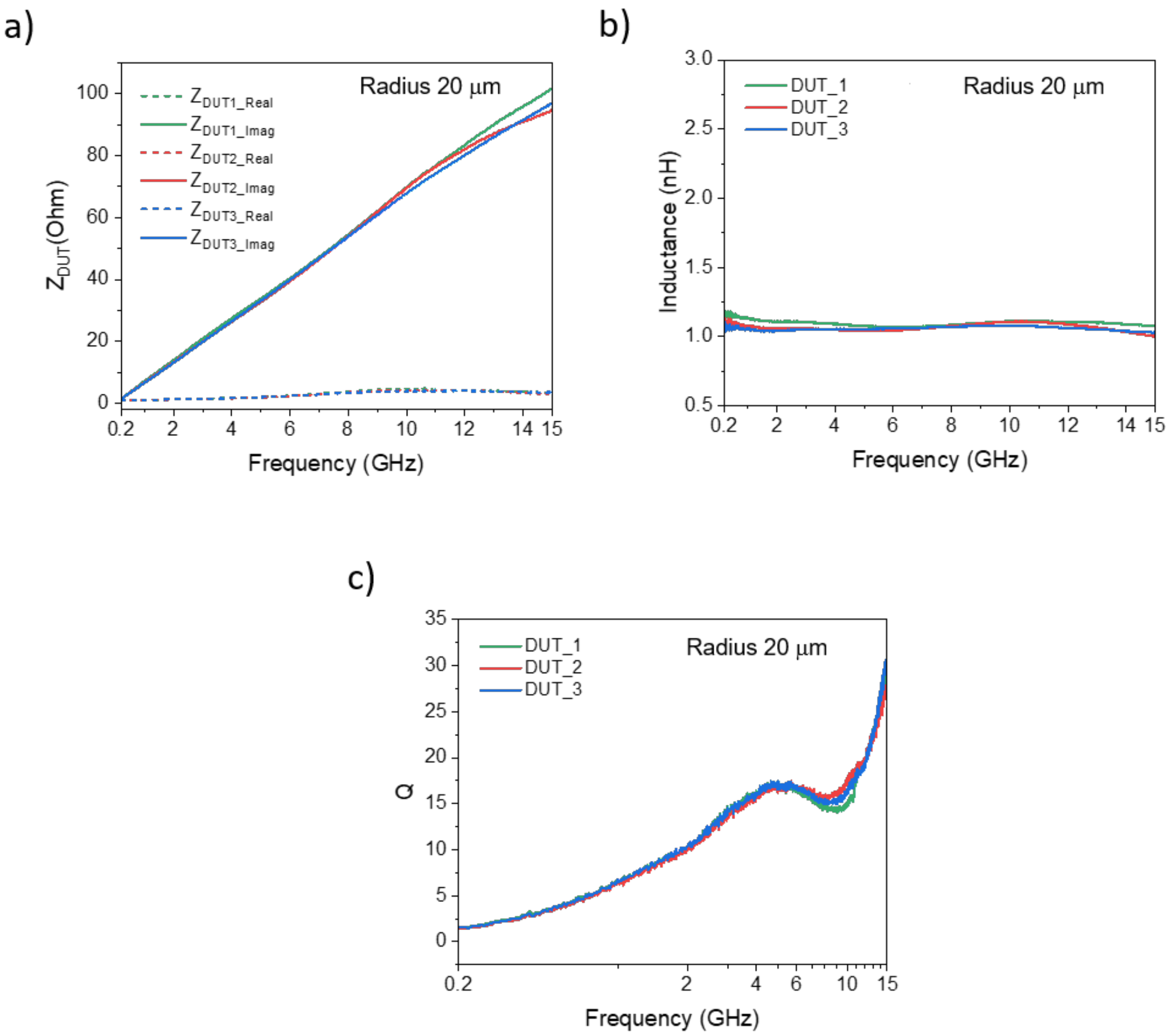

Figure S21. Characterization of printed microinductors up to 15 GHz. a) Frequency-resolved impedance spectra showing the real (dotted lines) and imaginary (solid lines) components of Z_{DUT} for three microinductors with radius of 20 μm, measured up to 15 GHz. b,c) Frequency-resolved inductance ($L=Imag(Z_{DUT})/\omega$) (b) and quality factor ($Q= Imag(Z_{DUT})/Real(Z_{DUT})$) (c) extracted from (a) over the range 0.2-15 GHz.

References

1. Ha, C. W., Prabhakaran, P. & Son, Y. 3D-printed polymer/metal hybrid microstructures with ultraprecision for 3D microcoils. *3D Print Addit Manuf* **6**, 165–170 (2019).
2. Nguyen, K. M. T., Yang, Z., Wang, A. T., Wicker, S. A. & Li, X. High-Frequency Inductors by Co-Design Optimization of Self-Rolled-up Membrane Technology. *Adv Electron Mater* https://doi.org/10.1002/aelm.202400639 (2025) doi:10.1002/aelm.202400639.
3. Huang, W. *et al. Monolithic Mtesla-Level Magnetic Induction by Self-Rolled-up Membrane Technology*. https://www.science.org (2020).
4. Xu, T. *et al.* 3D MEMS In-Chip Solenoid Inductor with High Inductance Density for Power MEMS Device. *IEEE Electron Device Letters* **40**, 1816–1819 (2019).
5. Le, H. T. *et al.* Fabrication of 3D air-core MEMS inductors for very-high-frequency power conversions. *Microsyst Nanoeng* **4**, (2018).
6. Wu, R. & Sin, J. K. O. A novel silicon-embedded coreless inductor for high-frequency power management applications. in *IEEE Electron Device Letters* vol. 32 60–62 (2011).
7. Kratt, K., Badilita, V., Burger, T., Korvink, J. G. & Wallrabe, U. A fully MEMS-compatible process for 3D high aspect ratio micro coils obtained with an automatic wire bonder. *Journal of Micromechanics and Microengineering* **20**, (2010).
8. Zia, M., Oh, H. & Bakir, M. S. Post-CMOS fabrication technology enabling simultaneous fabrication of 3-D solenoidal micro-inductors and flexible I/Os. *IEEE Trans Compon Packaging Manuf Technol* **8**, 2039–2044 (2018).
9. Kim, J., Kim, J. K., Kim, M., Herrault, F. & Allen, M. G. Microfabrication of toroidal inductors integrated with nanolaminated ferromagnetic metallic cores. *Journal of Micromechanics and Microengineering* **23**, (2013).
10. Gu, L. & Li, X. Concave-suspended high-$ Q $ solenoid inductors with an RFIC-compatible bulk-micromachining technology. *IEEE Trans Electron Devices* **54**, 882–885 (2007).
11. Liang, Y. C. *et al.* A concise process technology for 3-D suspended radio frequency micro-inductors on silicon substrate. *IEEE Electron Device Letters* vol. 23 700–703 Preprint at https://doi.org/10.1109/LED.2002.805748 (2002).
12. Meere, R. *et al.* Magnetic-core and air-core inductors on silicon: A performance comparison up to 100 MHz. *IEEE Trans Magn* **47**, 4429–4432 (2011).
13. Wang, X. N., Zhao, X. L., Zhou, Y., Dai, X. H. & Cai, B. C. Fabrication and performance of a novel suspended RF spiral inductor. *IEEE Trans Electron Devices* **51**, 814–816 (2004).
14. Burghartz, J. N. & Rejaei, B. On the design of RF spiral inductors on silicon. *IEEE Trans Electron Devices* **50**, 718–729 (2003).
