# Supplementary material for: On-chip 3D printing of metal microinductors for radio frequency electronics
Source: Nat Commun. 2026 Sep 8;17:10644. doi: 10.1038/s41467-026-77493-1 (PMC13649781; doi:10.1038/s41467-026-77493-1)
Supplement: Supplementary file 2 [file 41467_2026_77493_MOESM2_ESM.pdf]

nature portfolio

# Peer Review File

# On-Chip 3D Printing of Metal Microinductors for Radio Frequency Electronics

Corresponding Author: Dr Giuseppe Barillaro

This manuscript has been previously reviewed at another journal. This document only contains information relating to versions considered at Nature Communications.

**This file contains all reviewer reports in order by version, followed by all author rebuttals in order by version.**

Version 0:

Reviewer comments:

Reviewer #1

(Remarks to the Author)
The manuscript presents the fabrication and characterization of three-dimensional copper microinductors fabricated by localized electrodeposition and integrated directly onto chip-scale test structures. The revised manuscript includes additional measurements up to 15 GHz and more clearly articulates the proposed “zero-footprint” integration concept. The work has been improved compared with the previous version. However, several important issues remain insufficiently addressed.

Major Concerns

1. The significance of the proposed architectural concept remains insufficiently substantiated.

The manuscript is centered on the concept of a “zero-footprint” microinductor architecture, where freestanding inductors are printed above existing chip structures rather than occupying additional planar area. While this concept is repeatedly presented as the primary novelty and impact of the work, the manuscript does not convincingly demonstrate why this architectural change represents a substantial advance for RF integrated circuits.

In particular, the claimed benefits in terms of chip miniaturization, manufacturing cost reduction, and integration density remain largely qualitative. No circuit-level demonstration, quantitative area-cost analysis, or system-level evaluation is provided to establish the practical significance of the proposed approach. As a result, the manuscript asks the reader to accept the value of the architectural concept largely on the basis of intuition rather than evidence.

The authors should provide a more rigorous justification of the practical advantages of the proposed architecture, either through circuit-level demonstrations or through quantitative analysis showing how the reduction in planar area translates into meaningful benefits at the RF system level.

2. The “zero-footprint” terminology overstates the demonstrated benefit.

A central claim throughout the manuscript is the realization of “zero-footprint” inductors. However, the proposed structures do not eliminate physical footprint; rather, they relocate the inductor into the vertical dimension above the chip surface.

This distinction is important because the additional three-dimensional volume may introduce constraints associated with packaging, routing, shielding, mechanical robustness, thermal management, and manufacturing integration. These considerations are particularly relevant in RF systems, where packaging and electromagnetic coupling can strongly influence performance.

Given that these practical implications are not examined experimentally or quantitatively, the use of the term “zero footprint” appears overly strong. A more accurate description would be that the approach eliminates additional planar silicon area while introducing a three-dimensional structure above the chip surface. The manuscript should discuss this tradeoff more

explicitly and avoid implying that the proposed solution removes footprint-related constraints altogether.

3. The performance advantage relative to existing technologies remains unclear.

The reported inductance values (~0.8–3 nH) and quality factors (~11–17 in the GHz range) demonstrate successful operation. However, these values are not clearly superior to those achieved by existing integrated inductor technologies. The manuscript would benefit from a more comprehensive comparison with state-of-the-art approaches, including metrics such as inductance density, quality factor, occupied volume, fabrication complexity, integration compatibility, and current handling capability. Such analysis would help clarify the practical value and competitive position of the proposed approach.

4. High-frequency validation has improved but remains limited.

The addition of measurements up to 15 GHz significantly improves the manuscript. However, only a limited device geometry appears to have been characterized over this extended frequency range, while much of the reported characterization remains restricted to lower frequencies. As a result, some key claims regarding operation at substantially higher frequencies and self-resonance behavior continue to rely primarily on simulations. Additional broadband measurements across multiple device geometries would further strengthen the conclusions.

Based on these, the revised manuscript presents an interesting integration concept and demonstrates impressive fabrication capabilities. However, additional evidence is still needed to establish the practical significance and broader impact of the proposed architecture. Therefore, the reviewer recommends major revision.

Version 1:

Reviewer comments:

Reviewer #1

(Remarks to the Author)
The reviewer thanks the authors for their responses and revisions.
The manuscript has been improved and is suitable for publication.

**Open Access** This Peer Review File is licensed under a Creative Commons Attribution 4.0 International License, which permits use, sharing, adaptation, distribution and reproduction in any medium or format, as long as you give appropriate credit to the original author(s) and the source, provide a link to the Creative Commons license, and indicate if changes were made.
In cases where reviewers are anonymous, credit should be given to 'Anonymous Referee' and the source.
The images or other third party material in this Peer Review File are included in the article’s Creative Commons license, unless indicated otherwise in a credit line to the material. If material is not included in the article’s Creative Commons license and your intended use is not permitted by statutory regulation or exceeds the permitted use, you will need to obtain permission directly from the copyright holder.
To view a copy of this license, visit https://creativecommons.org/licenses/by/4.0/

Dear Reviewer,

We sincerely thank you for the careful evaluation of our manuscript entitled "On-Chip 3D Printing of Metal Microinductors for Radio Frequency Electronics" and for the constructive and insightful comments. We greatly appreciate the time and effort you devoted to reviewing our work.

Your suggestions have been important in strengthening the manuscript. In response, we have performed additional experiments, expanded the analysis, and substantially revised both the manuscript and the Supporting Information.

The principal changes include:

- extending the experimental high-frequency characterization up to 15 GHz for multiple microsolenoid geometries, providing broader experimental validation of the proposed architecture;
- strengthening the architectural significance of the proposed zero-footprint integration concept by quantitatively substantiating its implications for RF integrated circuits and clarifying the terminology throughout the manuscript;
- expanding the comparison with representative state-of-the-art technologies in Table S1 and revising the discussion to better position the proposed approach within the existing literature.

In addition, we have carefully revised the manuscript throughout to improve clarity, presentation, and consistency. A detailed point-by-point response to each of your comments is provided below, where all modifications are described and referenced.

We hope that the revised manuscript satisfactorily addresses your concerns and that you will find the manuscript significantly strengthened. We are grateful for your constructive feedback, which has substantially improved the quality and clarity of our work.

Sincerely,
Giuseppe Barillaro

**Reviewer's Comments**

The manuscript presents the fabrication and characterization of three-dimensional copper microinductors fabricated by localized electrodeposition and integrated directly onto chip-scale test structures. The revised manuscript includes additional measurements up to 15 GHz and more clearly articulates the proposed "zero-footprint" integration concept. The work has been improved compared with the previous version. However, several important issues remain insufficiently addressed.

**Comment: 1. The significance of the proposed architectural concept remains insufficiently substantiated.**
The manuscript is centered on the concept of a "zero-footprint" microinductor architecture, where freestanding inductors are printed above existing chip structures rather than occupying additional planar area. While this concept is repeatedly presented as the primary novelty and impact of the work, the manuscript does not convincingly demonstrate why this architectural change represents a substantial advance for RF integrated circuits.
In particular, the claimed benefits in terms of chip miniaturization, manufacturing cost reduction, and integration density remain largely qualitative. No circuit-level demonstration, quantitative area-cost analysis, or system-level evaluation is provided to establish the practical significance of the proposed approach. As a result, the manuscript asks the reader to accept the value of the architectural concept largely on the basis of intuition rather than evidence.
The authors should provide a more rigorous justification of the practical advantages of the proposed architecture, either through circuit-level demonstrations or through quantitative analysis showing how the reduction in planar area translates into meaningful benefits at the RF system level.

**Response**: We thank the reviewer for this important comment. We agree that the architectural significance of the proposed approach should be more explicitly justified. Accordingly, we have expanded both the Introduction and the Discussion, and revised Figure 1 caption to quantitatively illustrate the impact of eliminating the dedicated planar silicon area required by conventional on-chip inductors.
As discussed in the paper by Kang et al. [3] published in Nature Electronics (and thoroughly quantified in the related Supporting Information), planar inductors occupy 15% to 50% of the total die area in RF integrated circuits, depending on the technology node and circuit architecture. Specifically, inductors account for approximately 20% of the area in low-noise amplifiers (LNAs), 18% in band-pass filters, and up to 40% in transformer-based layouts. These values identify the inductor footprint as one of the principal limitations to further RF-IC scaling.

The architecture proposed in this work eliminates the need for additional dedicated planar silicon area by directly printing three-dimensional inductors above existing chip contact pads or across chip-to-package interconnects. Consequently, the silicon area conventionally occupied by planar inductors can instead be allocated to active circuitry, increased integration density, or reduced die size, depending on the specific circuit design. Since semiconductor manufacturing costs increase superlinearly with die area due to yield considerations, recovering even a fraction of the silicon area devoted to inductors has the potential to provide cost benefits in addition to increased functional density.

Although a complete RF circuit demonstration is beyond the scope of the present work, we believe that the quantitative analysis above, together with the revised Figure 1 and the expanded discussion in the manuscript, provides a more rigorous justification of the architectural advantages of the proposed approach for RF integrated circuits.

Action: We updated the Introduction section and Figure 1 quantifying the advantages of Si planar area reduction.
p.3 r.7-21 "Despite their importance, on-chip inductors represent one of the main bottlenecks in RF-IC design owing to their large footprint and limited scalability. Typical performance requirements include inductance values in the 1–10 nH range, quality factors (Q) exceeding 10 at the operating frequency, and self-resonance frequencies above 10 GHz[2]. Achieving these specifications inherently requires large planar structures, and inductors typically occupy 15–50% of the total die area, depending on the technology node and RF circuit architecture[3]. For example, inductors account for approximately 20% of the silicon area in low-noise amplifiers (LNAs), 18% in band-pass filters, and up to 40% in transformer-based layouts[3]. Since semiconductor fabrication cost increases superlinearly with die area due to yield consideration, the silicon area devoted to passive inductors has a disproportionate impact on both manufacturing cost and integration density. Unlike transistors and interconnects, which benefit from continuous downscaling, the footprint of conventional on-chip planar inductors is fundamentally constrained by electromagnetic considerations and therefore does not scale proportionally with CMOS technology nodes. Consequently, the inductor footprint has emerged as one of the principal barriers to further RF-IC miniaturization."

p.4 r.15-22 "Here, we introduce an architectural solution to the longstanding footprint limitation of planar inductors through the on-chip integration of three-dimensional copper microinductors for high frequency applications that require no additional planar silicon area, enabled by in-situ electrodeposition with sub-micrometer resolution. By directly printing freestanding metal-air microsolenoids onto chip contact pads (Figure 1b) or across chip-to-package interconnetcs (Figure 1c), the proposed approach decouples inductance from the planar silicon-area budget, enabling recovery of the silicon area conventionally occupied by inductors for increased integration density, additional active circuitry, or reduced die size."

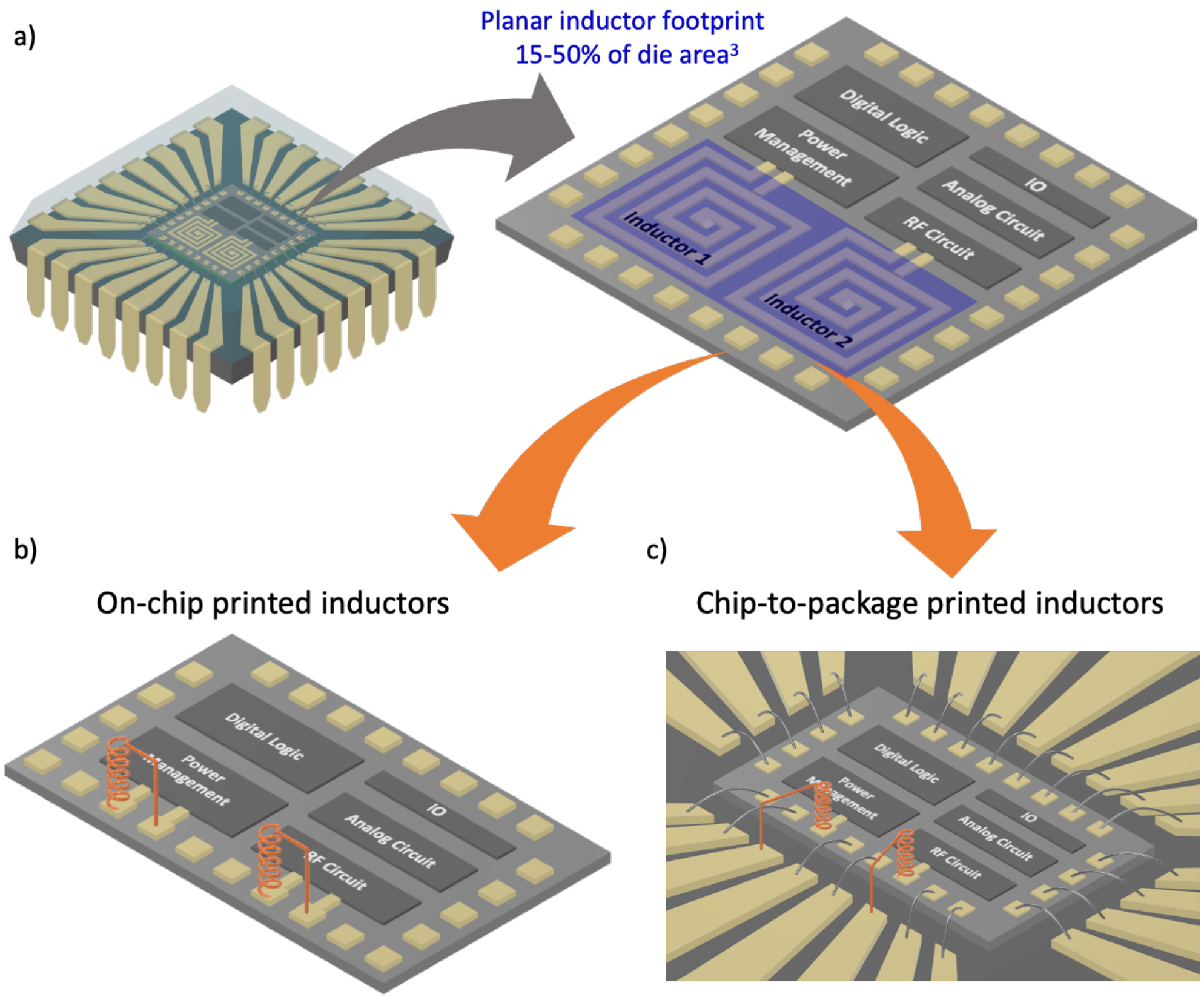


Figure 1: Concept of zero-footprint on-chip metal microinductors enabled by 3D printing. a) Conventional on-chip inductors occupy a significant portion of the planar silicon area in radio-frequency integrated circuits (RF-ICs), typically 15-50% of the total die area, depending on the chip architecture and technology node[3]). b,c) Three-dimensional microinductors are directly printed onto chip contact pads (b) or across chip-to-package interconnects (c), eliminating the need for additional dedicated planar silicon area. This architecture has the potential to recover the 15–50% of the silicon area typically occupied by planar inductors, enabling increased integration density, additional active circuitry, or reduced die size, depending on the RF-IC design."

**Comment: 2. The "zero-footprint" terminology overstates the demonstrated benefit.**
A central claim throughout the manuscript is the realization of "zero-footprint" inductors. However, the proposed structures do not eliminate physical footprint; rather, they relocate the inductor into the vertical dimension above the chip surface.
This distinction is important because the additional three-dimensional volume may introduce constraints associated with packaging, routing, shielding, mechanical robustness, thermal management, and manufacturing integration. These considerations are particularly relevant in RF systems, where packaging and electromagnetic coupling can strongly influence performance.
Given that these practical implications are not examined experimentally or quantitatively, the use of the term "zero footprint" appears overly strong. A more accurate description would be that the approach eliminates additional planar silicon area while introducing a three-dimensional structure above the chip surface. The manuscript should discuss this tradeoff more explicitly and avoid implying that the proposed solution removes footprint-related constraints altogether.

**Response**: We thank the reviewer for raising this important point. We agree that the term zero-footprint should be interpreted precisely. In the revised manuscript, we clarify that footprint refers specifically to the additional dedicated planar silicon area required by conventional on-chip inductors. The proposed inductors do not eliminate the physical volume of the component; rather, they relocate the inductive element into the third dimension above existing chip contact pads or across chip-to-package interconnects, thereby eliminating the need for additional planar silicon area.
The architectural advantage therefore lies in decoupling inductance from the planar silicon-area budget, rather than eliminating the physical size of the inductor. This concept is now emphasized throughout the revised manuscript and in Figure 1. It is further supported by the experimental and simulated results: for a fixed coil radius (i.e., fixed planar footprint), increasing the number of turns increases inductance by exploiting the vertical dimension (Figure 2d and Figure S16f). These results demonstrate that the vertical dimension provides an additional degree of freedom to increase inductance without increasing the planar silicon area required by the device.

We also agree that the three-dimensional geometry introduces practical considerations related to packaging, routing, shielding, thermal management, mechanical robustness, and electromagnetic coupling. We have therefore expanded the Conclusions to explicitly acknowledge these trade-offs and clarify that the proposed architecture removes the requirement for additional dedicated planar silicon area but does not eliminate the need to address package- and circuit-level integration. Importantly, this architectural concept is consistent with the evolution of modern advanced semiconductor packaging, where advanced technologies such as wafer-level packaging, fan-out packaging, redistribution-layer architectures, and system-in-package integration routinely integrate non-planar and vertically interconnected structures to increase functional density while minimizing planar area [27].
As an initial step toward package compatibility, we demonstrate PDMS encapsulation of the printed microinductors (Figure S19), which does not produce measurable changes in the impedance spectra or extracted lumped parameters, indicating that mechanical encapsulation can be achieved without degrading electrical performance in the investigated frequency range.
We further clarify in the Conclusions section that comprehensive package-level reliability, thermal management, electromagnetic compatibility, and circuit-specific integration studies remain beyond the scope of the present work and will be addressed in future RF-IC demonstrations.

Action: We updated the Results and Discussion and Conclusions sections, clarifying the zero-footprint terminology and the benefits of the silicon planar area reduction.

p.5 r.16-18 - p.6 r. 1-4 "We first conducted three-dimensional full-wave electromagnetic simulations to define the design space of the microsolenoids and investigate how the third dimension can be exploited to increase inductance and quality factor without increasing the dedicated planar silicon area required by conventional on-chip inductors. The simulations evaluate the frequency-dependent inductive behavior as a function of key geometrical parameters, namely coil radius, number of turns, and coil density, to identify design strategies for maximizing RF performance while preserving a compact planar footprint and to guide fabrication and characterization."

p.7 r.12-14 "For a fixed coil radius (i.e., fixed planar footprint), the microsolenoid design exploits the third dimension to optimize inductive performance without increasing the dedicated planar area."

p.7 r.25 - p.8 r. 1-10 "Increasing the coil radius to 40 µm while keeping the number of turns and coil density unchanged increases $L_{Q_{max}}$ to approximately 1.63 $nH$, with comparable $Q_{max}$ of about 6, although the peak shifts to a lower frequency of approximately 50 GHz. Alternatively, maintaining the same 40 µm radius (i.e., the same planar footprint) and increasing the coil density from 5 to 10 turns per 100 µm further increases $L_{Q_{max}}$ to approximately 2.3 $nH$ while increasing $Q_{max}$ to about 80, with only a modest reduction in peak frequency to about 40 GHz.

This comparison highlights that, unlike conventional planar inductors where higher inductance is primarily achieved by increasing the footprint, the proposed microsolenoid architecture exploits the third dimension to further enhance inductive performance while preserving the planar footprint area."

p.17 r.23-24 p.18 r.1-16 "In conclusion, on-chip inductors remain a major bottleneck in radio frequency integrated circuits (RF-ICs) because to their large planar footprint and limited scalability constrain miniaturization, integration density, and manufacturing cost.
Here, we introduced an architectural solution to this longstanding limitation by enabling three-dimensional copper microinductors that require no additional dedicated planar silicon area. Using in-situ electrodeposition with sub-micrometer resolution, freestanding metal–air microsolenoids were directly printed on measurement chip, exploiting the third dimension as an additional degree of freedom for RF inductor design. The printed microinductors achieved inductance values in the nH range and quality factors up to 18 in the GHz regime, in agreement with full-wave electromagnetic simulations, with inductive behavior experimentally validated up to 15 GHz.
By decoupling inductance from the planar silicon-area budget, this approach enables increased inductive performance through three-dimensional design rather than increased silicon footprint. The architecture is conceived as a post-fabrication integration strategy compatible with modern advanced semiconductor packaging technologies—including wafer-level packaging, fan-out packaging, redistribution-layer architectures, and system-in-package integration—which routinely accommodate non-planar structures and three-dimensional interconnects.[27] At the same time, practical aspects such as package-level reliability, thermal management, and circuit-specific electromagnetic compatibility remain important considerations and will require dedicated investigation in future integrated RF-IC demonstrations."

**3. The performance advantage relative to existing technologies remains unclear.**
The reported inductance values (~0.8–3 nH) and quality factors (~11–17 in the GHz range) demonstrate successful operation. However, these values are not clearly superior to those achieved by existing integrated inductor technologies. The manuscript would benefit from a more comprehensive comparison with state-of-the-art approaches, including metrics such as inductance density, quality factor, occupied volume, fabrication complexity, integration compatibility, and current handling capability. Such analysis would help clarify the practical value and competitive position of the proposed approach.
Response: We thank the reviewer for this valuable suggestion. A comprehensive comparison with representative state-of-the-art air-core integrated inductor technologies was already provided in

Table S1 of the Supporting Information, including fabrication technology, device geometry, inductance, quality factor, self-resonance frequency, planar footprint, and compatibility with on-chip integration. To address the reviewer's comment more explicitly, we have expanded Table S1 and the discussion in the manuscript to better highlight the competitive position of the proposed approach.

As correctly noted by the reviewer, the proposed microinductors are not intended to maximize inductance or quality factor beyond the current state of the art. Rather, they achieve RF performance comparable to existing integrated technologies while eliminating the requirement for additional dedicated planar silicon area, which represents the principal architectural advance of this work. Unlike conventional approaches, where increased inductance generally requires increased silicon footprint, the proposed three-dimensional architecture relocates inductors on existing pads, exploits the vertical dimension to preserve the planar silicon area while maintaining competitive inductance and quality-factor values.

Table S1 further shows that the proposed technology combines competitive inductance and quality factor with zero additional planar footprint, direct post-fabrication integration on existing chip pads or interconnects, and predicted self-resonance frequencies extending well beyond the experimentally investigated frequency range. We have revised the manuscript to emphasize that these characteristics collectively define the practical value and unique competitive position of the proposed approach.

Action: We updated the Introduction and Results and Discussion sections to include a direct comparison of the proposed approach with the state of the art.

p.4 r.2-14 "As summarized in Table S1, these approaches achieve different trade-offs between inductance, quality factor, fabrication complexity, and footprint. While many improve inductance density or quality factor, they generally preserve the fundamental trade-off between inductive performance and dedicated planar silicon area, and often require increasingly complex fabrication processes.
In this context, additive manufacturing techniques, such as 3D printing, offer opportunities to realize three-dimensional structures beyond the constraints of planar fabrication[14,15]. Notable approaches include two-photon polymerization[16], multi-photon lithography[17], and ice lithography[18]. However, most high-resolution techniques rely on polymer-based processes that require subsequent metallization, limiting conductivity, scalability, and compatibility with standard microelectronic platforms. As further highlighted in Table S1, direct 3D printing of fully metallic on-chip microinductors has remained largely unexplored despite its potential to decouple inductive performance from the planar silicon-area budget."

p.17 r.7-19 "A comparison with representative state-of-the-art air-core integrated inductor technologies is provided in Table S1, which summarizes their fabrication approach, geometry, inductance, quality factor, self-resonance frequency, footprint, and integration compatibility. The comparison shows that existing technologies achieve different trade-offs between electrical performance, footprint, and fabrication complexity. While some approaches report higher inductance or quality factor, these improvements are generally obtained at the expense of increased dedicated planar silicon area, more complex fabrication processes, or reduced compatibility with post-fabrication integration. In contrast, the present approach combines competitive RF performance with no additional dedicated planar silicon area, achieved by enabling direct printing

freestanding microsolenoids onto existing chip contact pads or interconnects. By exploiting the third dimension rather than increasing the planar footprint, this architecture decouples inductive performance from the planar silicon-area budget, providing a distinctive design strategy for future RF integrated circuits while remaining fully compatible with post-CMOS integration."

**4. High-frequency validation has improved but remains limited.**

The addition of measurements up to 15 GHz significantly improves the manuscript. However, only a limited device geometry appears to have been characterized over this extended frequency range, while much of the reported characterization remains restricted to lower frequencies. As a result, some key claims regarding operation at substantially higher frequencies and self-resonance behavior continue to rely primarily on simulations. Additional broadband measurements across multiple device geometries would further strengthen the conclusions.

Response: We thank the reviewer for emphasizing the importance of broadband experimental validation. We agree that additional measurements on multiple device geometries would further strengthen the manuscript. Accordingly, we have expanded the high-frequency measurements by characterizing additional microsolenoids with different coil radii up to 15 GHz (new Figure 4o-q). These measurements confirm that the experimentally observed trends in inductance and quality factor are consistently preserved across different device geometries, providing broader experimental validation of the proposed architecture.

However, the upper frequency range of the present experimental platform is fundamentally limited by the dielectric losses and parasitic response of the FR4-based gap-interrupted coplanar waveguide (giCPW) used for electrical characterization, rather than by the printed microinductors themselves. For this reason, we continue to distinguish throughout the manuscript between the experimentally validated performance and the intrinsic high-frequency behavior predicted by full-wave electromagnetic simulations.

We have clarified this point in the Conclusions section of the revised manuscript and explicitly state that future characterization using dedicated low-loss RF substrates and custom integrated test platforms will enable experimental validation over a substantially broader frequency range.

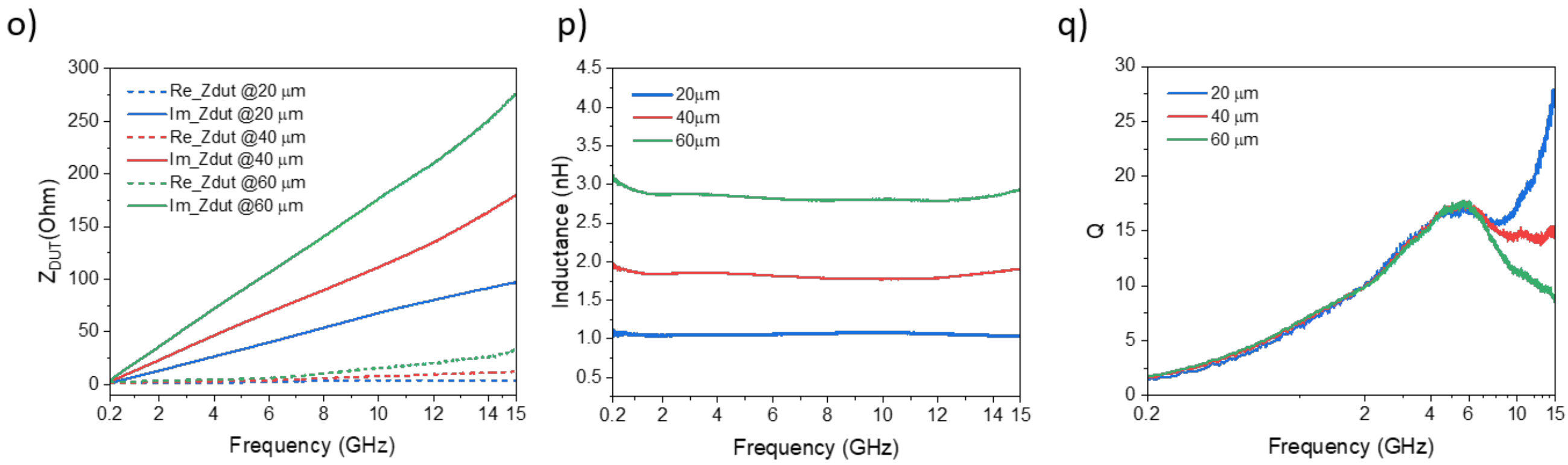


Figure 4. o) Frequency-resolved impedance spectra showing the real (dotted lines) and imaginary (solid lines) components of ZDUT for microinductors with radius of 20,40, and 60 mm, measured up to 15 GHz. p,q) Frequency-resolved inductance (L=Imag(ZDUT)/w) and quality factor (Q= Imag(ZDUT)/Real(ZDUT)) extracted from (o) over the range 0.2-15 GHz.

Action: We performed additional experiments regarding the high-frequency behavior of microcoils with different radii and updated Figure 4, the Results and Discussion and the Conclusion sections accordingly.

p.16, r.23-25, p.17, r.1-9: Finally, the high-frequency response of the microsolenoids with different radii from 20 to 60 mm was investigated up to 15 GHz (Figure 4o–q). Experimental measurements indicate excellent reliability of the high-frequency behavior over multiple devices (Figures S20,21). The impedance spectra retain predominantly inductive behavior over the extended frequency range regardless of the microsolenoid radius, with the imaginary component increasing linearly with frequency, while the real component remains relatively low and exhibits a gradual increase due to frequency-dependent resistive losses (Figure 4o). The inductance scales with the radius of the microsolenoids, remaining approximately constant over frequency and in agreement with the low frequency values, while the quality factor increases up to ~18 at ~5 GHz (Figure 4p,q). The non-monotonic evolution of Q highlights that the upper frequency range is fundamentally limited by the dielectric losses and parasitic response of the FR4-based gap-interrupted coplanar waveguide used for electrical characterization, rather than by the printed microinductors themselves. These results demonstrate that the printed microinductors preserve inductive operation well into the multi-GHz regime.

p.4 r.2-14 "Future work will focus on extending operation to higher frequencies through the use of low-loss substrates and custom IC platforms, as well as further improving performance through optimization of microsolenoid geometry, incorporation of functional magnetic materials, and enhancement of the metal printing process to reduce resistive losses and increase conductivity."
